# PARP1-PKM2 Axis Mediates Right Ventricular Failure Associated With Pulmonary Arterial Hypertension

**DOI:** 10.1016/j.jacbts.2022.01.005

**Published:** 2022-03-16

**Authors:** Tsukasa Shimauchi, Olivier Boucherat, Tetsuro Yokokawa, Yann Grobs, WenHui Wu, Mark Orcholski, Sandra Martineau, Junichi Omura, Eve Tremblay, Kana Shimauchi, Valérie Nadeau, Sandra Breuils-Bonnet, Roxane Paulin, François Potus, Steeve Provencher, Sébastien Bonnet

**Affiliations:** a Pulmonary Hypertension Research Group, Centre de Recherche de l’Institut Universitaire de Cardiologie et de Pneumologie de Québec, Québec, Québec, Canada; b Department of Medicine, Université Laval, Québec, Québec, Canada

**Keywords:** PARP1, PKM2, pulmonary hypertension, right ventricle, cKO, conditional knockout, CM, cardiomyocyte, CO, cardiac output, ET, endothelin, NF-κB, nuclear factor κB, PAB, pulmonary artery banding, PAH, pulmonary arterial hypertension, PARP1, poly(adenosine diphosphate–ribose) polymerase 1, PKM2, pyruvate kinase muscle isozyme 2, RV, right ventricular, STAT3, signal transducer activator of transcription 3, WT, wild-type

## Abstract

The authors show that increased poly(adenosine diphosphate–ribose) polymerase 1 (PARP1) and pyruvate kinase muscle isozyme 2 (PKM2) expression is a common feature of a decompensated right ventricle in patients with pulmonary arterial hypertension and animal models. The authors find in vitro that overactivated PARP1 promotes cardiomyocyte dysfunction by favoring PKM2 expression and nuclear function, glycolytic gene expression, activation of nuclear factor κB–dependent proinflammatory factors. Pharmacologic and genetic inhibition of PARP1 or enforced tetramerization of PKM2 attenuates maladaptive remodeling improving right ventricular (RV) function in multiple rodent models. Taken together, these data implicate the PARP1/PKM2 axis as a critical driver of maladaptive RV remodeling and a new promising target to directly sustain RV function in patients with pulmonary arterial hypertension.

## Highlights

• In PAH, PARP1 promotes cardiomyocyte dysfunction by impacting PKM2/NF-kB axis
• Pharmacological inhibition of PARP1/activation of PKM2 prevent RV dysfunction in PAB rats
• Global inactivation of Parp1 confers protection against PAB-induced RV dysfunction
• Cardiomyocyte-targeted inactivation of Parp1 attenuates Su/Hx-induced RV dysfunction
• Targeting PARP1/PKM2 may represent a promising avenue to support RV function in PAH

## Visual Abstract

**Figure:**
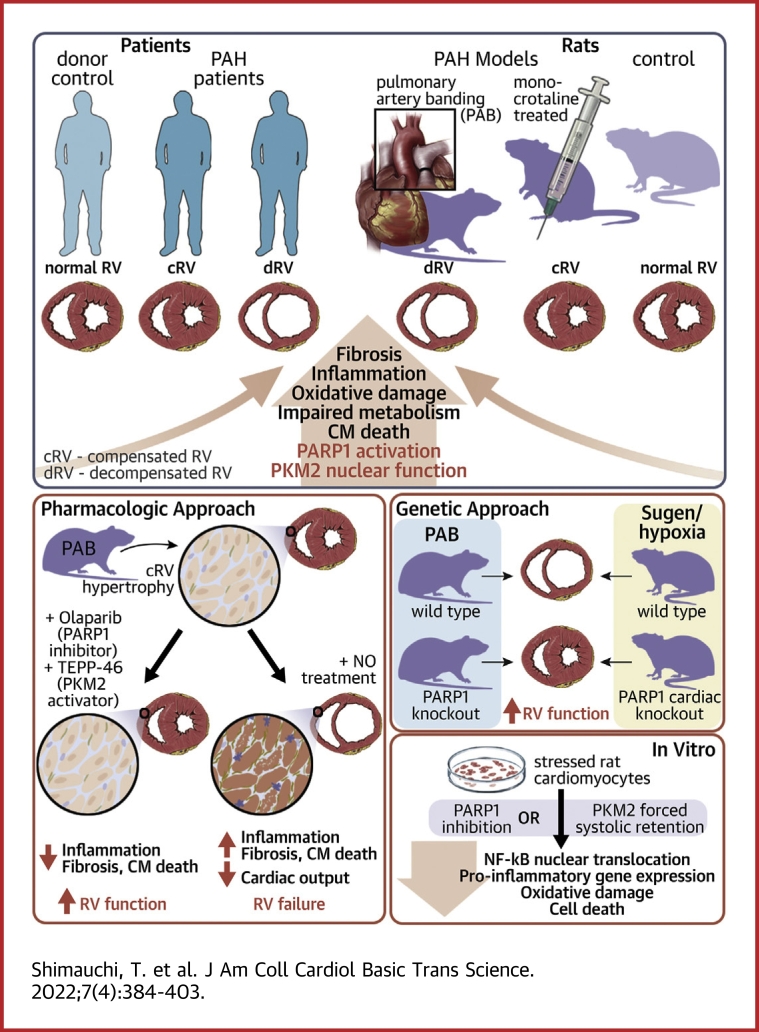

Pulmonary arterial hypertension (PAH) is a life-threatening condition characterized by a progressive obstruction of small caliber pulmonary arteries caused by sustained vasoconstriction and extensive vascular remodeling.^1^^,^^2^ The narrowing of pulmonary arteries is accompanied by a gradual increase in pulmonary vascular resistance and mean pulmonary artery pressure, pushing the right ventricle to undergo structural and functional changes, a process typically divided into 2 stages.^3^, ^4^, ^5^ In the early stage, right ventricular (RV) remodeling is marked by concentric hypertrophy, boosting contractility and preserving adequate cardiac output (CO) in the face of increased afterload. The adaptive capacity of the right ventricle is limited, and this “compensated” state inexorably transitions toward a maladaptive form (decompensated stage) with rapid RV dilatation, decreased contractility, drop of CO, and ultimately death.^4^^,^^6^, ^7^, ^8^ Although RV function represents the major determinant of functional capacity and prognosis in patients with PAH, it has long been considered an “innocent bystander.” However, on the basis of clinical evidence showing that approved drugs for PAH treatment, which primarily address the vasoconstrictive phenotype of pulmonary artery cells, fail to significantly improve long-term survival^9^ and that patients with PAH experiencing persistently impaired RV function despite improvements in pulmonary hemodynamic status continue to have a poor prognosis,^10^ a large amount of research is now directed toward a better understanding of the disease-related mechanisms contributing to pathological remodeling, with the overarching goal of simultaneously tackling maladaptive remodeling of the right ventricle and the pulmonary circulation.

Supported by large-scale omics analysis, the available evidence indicates that alterations in energy metabolism (the switch from fatty acid oxidation to glycolytic carbohydrate metabolism), increased oxidative stress, enhanced inflammation, fibrosis, and cardiomyocyte (CM) death contribute to a self-perpetuating vicious cycle of tissue damage culminating in RV failure.^4^^,^^8^^,^^11^ Given the complexity and multifactorial nature of pathological RV remodeling in PAH, it is of great interest to identify new actionable targets that intersect with the multiple detrimental cellular stress responses triggering maladaptive RV remodeling, especially for pathways concomitantly involved in pulmonary artery remodeling. This is a difficult task, as most antiremodeling drugs targeting the pulmonary vasculature have revealed no direct effect or even negative effects on the stressed right ventricle.^12^

Interestingly, pyruvate kinase muscle isozyme 2 (PKM2), already known to contribute to pulmonary artery obliteration,^13^ was identified as a molecular integrator of anaerobic metabolism, oxidative stress, tissue inflammation, and fibrosis in several diseases sharing pathogenic similarities with RV failure.^14^, ^15^, ^16^ Expression and activity of PKM2 are subject to multiple layers of regulation. Among them, PKM2 oscillates between a low-activity dimer form and a highly active tetramer form. In its metabolic role, the dimeric form of PKM2 catalyzes the final and rate-limiting step of glycolysis, while tetrameric PKM2 promotes adenosine triphosphate production through the tricarboxylic acid cycle.^17^ Beyond its canonical enzymatic function, dimeric PKM2 can also translocate to the nucleus, where it acts as a transcriptional coactivator enhancing the activity of multiple transcription factors, such as hypoxia-inducible factor 1α,^18^^,^^19^ nuclear factor κB (NF-κB),^20^ and signal transducer activator of transcription 3 (STAT3),^21^ leading to the up-regulation of proglycolytic (glucose transporter 1, lactate dehydrogenase, hexokinase 2) and proinflammatory/pro-fibrotic (*IL-1β*, *IL-6*, *IL-8*, and *CCL2*) genes.^14^^,^^19^^,^^22^^,^^23^ Interestingly, the nuclear retention and functions of PKM2 have been documented to be dependent on poly(adenosine diphosphate–ribose) polymerase 1 (PARP1),^23^ for which the inhibition was also previously documented to reverse pulmonary vascular remodeling in patients with PAH.^24^ Although the PARP1/PKM2 axis appears to be multitasking stress modulator closely involved in pathological cardiac remodeling, their direct implication in RV failure has been insufficiently investigated.

In the present study, we provide evidence that increased PARP1 and PKM2 expression is a common feature of a decompensated right ventricle in patients with PAH and animal models of RV failure. Using a pharmacologic, molecular, and genetic approach, we demonstrate in vitro that PARP1 and the associated nuclear PKM2 fuel a vicious cycle of metabolic, oxidative DNA damage and inflammatory disturbances, causing CM dysfunction and death. Using the pulmonary artery banding (PAB) model, which offers the advantage of examining RV responses to pressure overload as well as interrogating the impacts of putative therapies without the confounding effects of the pulmonary vasculature, we also demonstrate that pharmacologic and genetic inhibition of PARP1 and enforced tetramerization of PKM2 significantly attenuate the typical hallmarks of maladaptive remodeling and directly improve RV function. Finally, we show that mice with CM-restricted inactivation of *Parp1* also exhibit RV structural and functional improvements when subjected to increased afterload.

## Methods

For experimental methods describing in vitro and cellular assays and detailed in vivo methods, refer to the Supplemental Appendix.

### Human tissue samples

All experimental procedures using human tissues or cells conformed to the principles outlined in the Declaration of Helsinki and were performed with the approval of Laval University and the Institut Universitaire de Cardiologie et de Pneumologie de Québec Biosafety and Ethics Committees (CER #20773, CER #20735, and CER #21747). All experiments were performed in accordance with the latest preclinical PAH research guidelines.^25^ Tissues were obtained from patients who had previously given written informed consent. RV samples were categorized as control, compensated, and decompensated right ventricles on the basis of clinical history and cardiac index. In brief, control right ventricle was obtained from patients with normal RV function who underwent cardiac surgery or early autopsies of patients who did not have any cardiac or respiratory diseases. Compensated right ventricle was obtained from cardiac biopsies or autopsies of patients with RV hypertrophy, preserved cardiac index (>2.2 L/min/m^2^) or normal tricuspid annular plane systolic excursion measured using echocardiography. Decompensated right ventricle was obtained from early autopsies of patients with end-stage PAH. Clinical characteristics are described in Supplemental Tables 1 and 2.

### Animal studies

All animal experiments were performed according to the guidelines of the Canadian Council on Animal Care and approved by the institutional animal care and use committees of University Laval and the Québec Heart and Lung Institute Research Centre (#2018-015-3).

### Statistical analysis

Values are expressed as mean ± SEM. The Shapiro-Wilk test was performed to determine if the data followed a normal distribution. Comparisons of means between 2 groups were performed using unpaired Student’s *t-test* and the Mann-Whitney *U* test for normally and not normally distributed samples, respectively. Comparisons of means among 3 or more groups were performed using 1-way analysis of variance for normally distributed samples, followed by the Tukey honestly significant difference method for multiple pairwise comparisons. Comparison among multiple groups for non-normally distributed data were performed using the nonparametric Kruskal-Wallis test. For real-time polymerase chain reaction measurements with skewed data, a log transformation was applied. All analyses were conducted using GraphPad Prism version 9 (GraphPad Software). *P* values <0.05 were considered to indicate statistical significance.

## Results

### Up-regulation of the PARP1/PKM2 axis in decompensated right ventricle from patients with PAH and animal models

**Figure 1:**
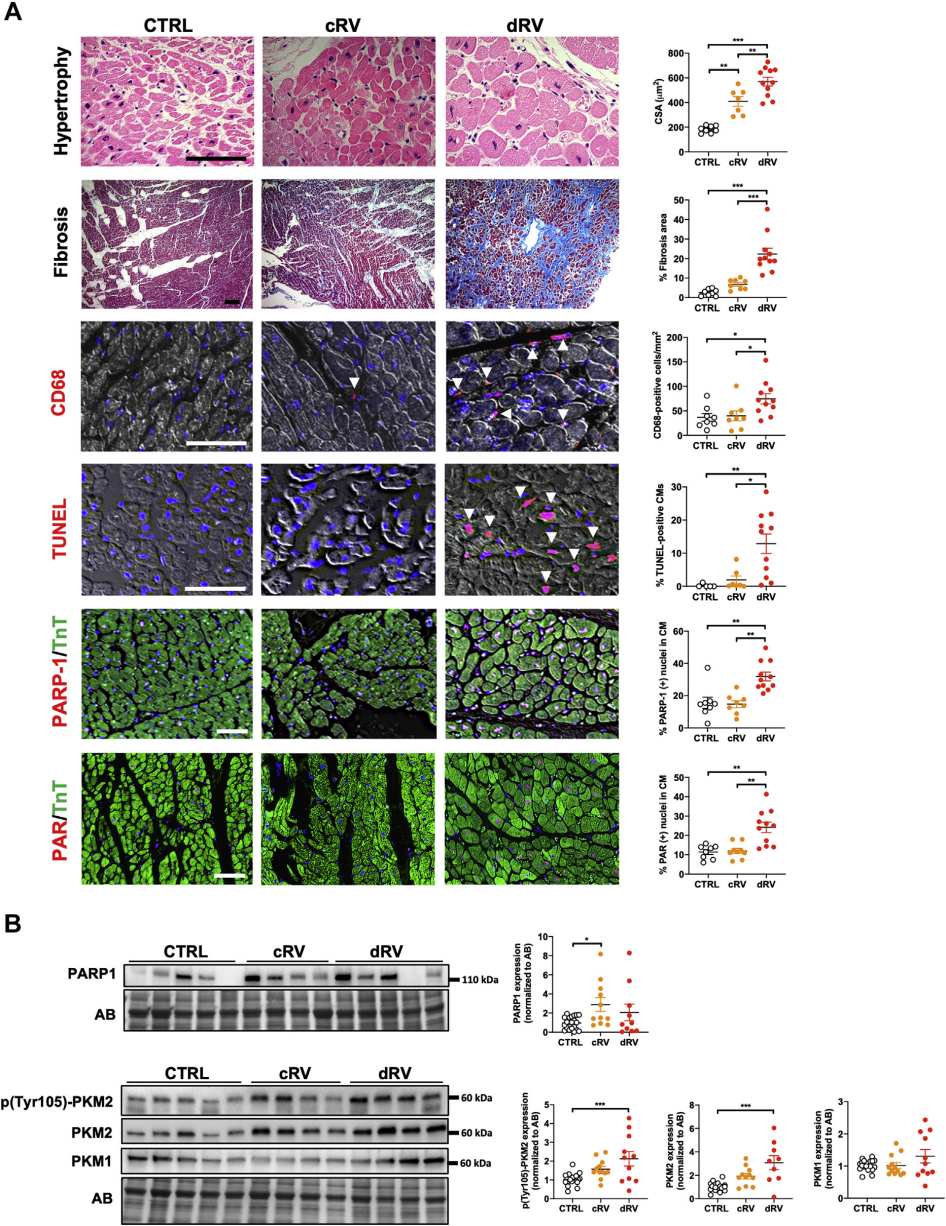
Increased Expression and Activity of PARP1 and PKM2 in dRV From Patients With PAH **(A)** Representative images and corresponding quantification of human right ventricular (RV) sections from control subjects (CTRL) (n = 5-8), compensated right ventricle (cRV; n = 7 or 8), and decompensated right ventricle (dRV; n = 11) patients with PAH stained with hematoxylin and eosin (cardiomyocyte surface area [CSA]), Masson’s trichrome (fibrosis), cluster of differentiation 68 (CD68) (infiltration of macrophages), or terminal deoxynucleotidyl transferase dUTP nick-end labeling (TUNEL; apoptosis) or double-labeled for troponin T (TnT) and either poly(adenosine diphosphate–ribose) polymerase 1 (PARP1) or poly(adenosine diphosphate–ribose) (PAR). **(B)** Representative western blots and quantification of PARP1, phosphorylated pyruvate kinase muscle isozyme 2 (pPKM2), PKM2, and pyruvate kinase muscle isozyme 1 (PKM1) in human right ventricle from control subjects (n = 20), cRV (n = 11), and dRV (n = 10 or 11) patients with PAH. Scale bars, 50 mm. **Arrowheads** denote positive cells. ∗*P <* 0.05, ∗∗*P <* 0.01, and ∗∗∗*P <* 0.001. Scatter dot plots show individual values and mean ± SEM. Comparisons were made using 1-way analysis of variance followed by Tukey multiple-comparison tests. AB = amido black; CM = cardiomyocyte.

**Figure 2:**
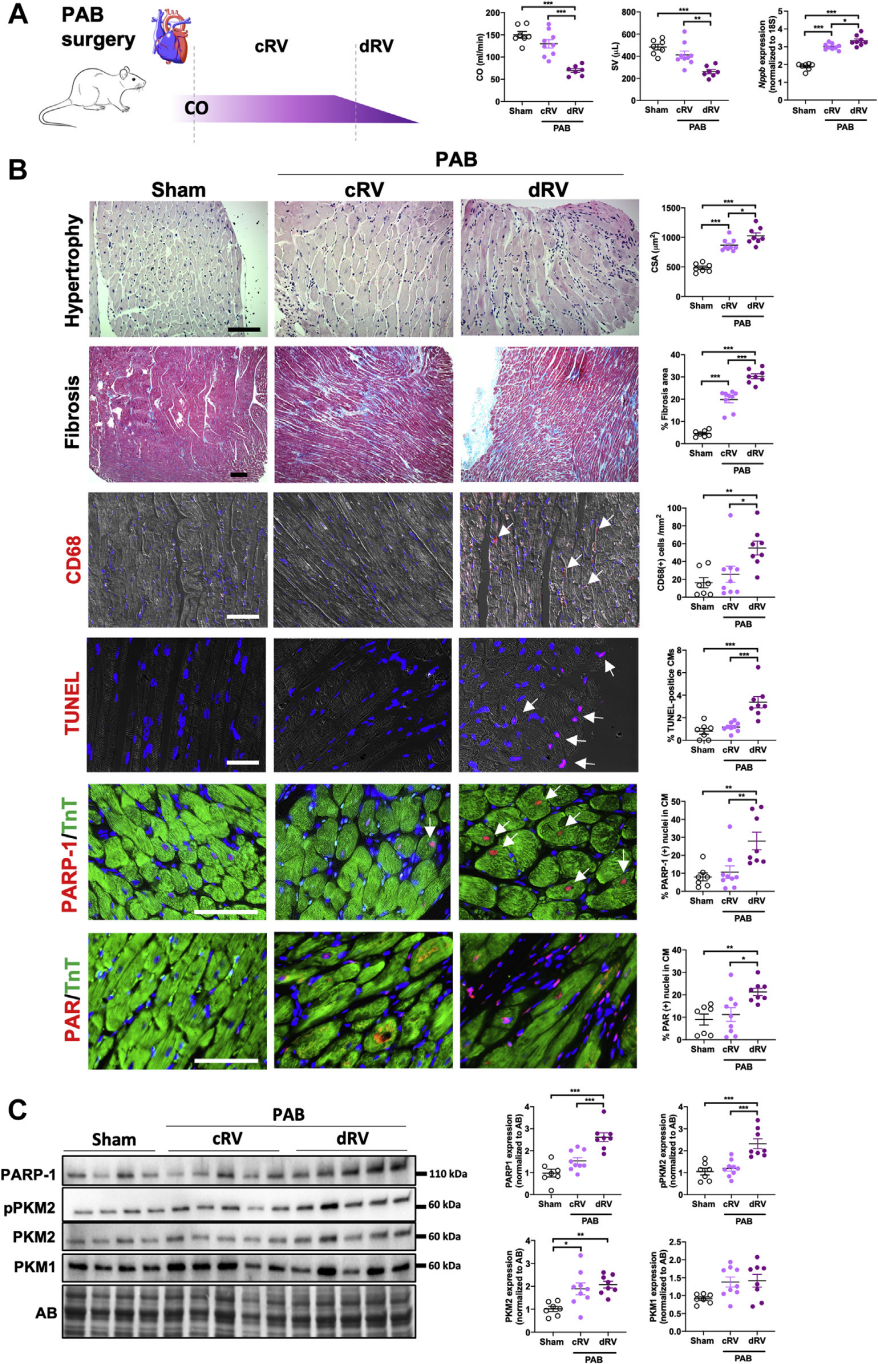
Increased Expression and Activity of PARP1 and PKM2 in Decompensated Right Ventricles From PAB Rats **(A)** Schematic representation of categorization of pulmonary artery banding (PAB) rats on the basis of hemodynamic status assessed using echocardiography and relative messenger RNA levels of *Nppb*. PAB rats underwent right heart catheterization at different time points over a period of 6 weeks, starting 2 weeks after PAB surgery. A group of sham-operated rats was used to define normal cardiac index (CO) and stroke volume (SV). PAB rats were subsequently classified into cRV or dRV groups on the basis of hemodynamic data. The relative expression levels of *Nppb* were used to validate the classification. **(B)** Representative images and corresponding quantification of RV sections from sham-operated (n = 7) and PAB-operated rats classified as cRV (n = 9) or dRV (n = 8) stained with hematoxylin and eosin (cardiomyocyte cell surface area), Masson’s trichrome (fibrosis), CD68 (infiltration of macrophages), or TUNEL (apoptosis) or double-labeled for TnT and either PARP1 or PAR. **(C)** Representative western blots and quantification of PARP1, pPKM2, PKM2, and PKM1 in right ventricles from rats subjected to PAB or sham surgery. Sham, n = 7; cRV, n = 9; dRV, n = 8. Scale bars, 50 mm. **Arrowheads** denote positive cells. ∗*P <* 0.05, ∗∗*P <* 0.01, and ∗∗∗*P <* 0.001. Scatter dot plots show individual values and mean ± SEM. Comparisons were made using 1-way analysis of variance followed by Tukey multiple-comparison tests or nonparametric Kruskal-Wallis tests. Abbreviations as in Figure 1.

Before examining the expression levels of PARP1 and PKM2 in humans and rodents (subjected to monocrotaline injection and PAB), RV samples, hemodynamically categorized as normal, compensated, or decompensated as previously done^26^ (Supplemental Tables 1 to 3, Figure 2A, Supplemental Figure 1A), were characterized at the histologic and biochemical levels. Not surprisingly, RV CMs demonstrated progressive hypertrophy over the remodeling process (Figures 1A and 2B, Supplemental Figure 1B). Fibrosis and inflammation, respectively assessed using Masson’s trichrome stain and recruitment of cluster of differentiation 68–positive macrophages, were minimal in compensated right ventricle to significantly increased in decompensated right ventricle (Figures 1A and 2B, Supplemental Figure 1B). Expression levels of the activated/phosphorylated form of STAT3 (a promoter of cardiac inflammation^27^) and associated proinflammatory factors, including interleukin-8 (*IL-8*) and C-C motif chemokine ligand 2 (*Ccl2*), exhibited the same pattern (Supplemental Figures 2 and 3). Similarly, expression levels of the transcription factor cMYC, an important promoter of glycolysis, and the glycolytic enzymes hexokinase 2 and lactate dehydrogenase were significantly augmented in decompensated right ventricle (Supplemental Figures 2 and 3). Furthermore, the expression of Nudix hydrolase 1 (a nucleotide poll sanitizing enzyme up-regulated in response to oxidative insult)^28^ and CM apoptosis (quantified by terminal deoxynucleotidyl transferase dUTP nick-end labeling staining) were specifically increased in decompensated right ventricle (Figures 1A and 2B, Supplemental Figures 1B and 2A to 2C). As human and rodent RV samples fulfill the standard hemodynamic, histologic, and biochemical criteria of healthy, adaptive, and maladaptive remodeling, we next measured PARP1 and PKM2 in this tissue. As shown in Figure 1, immunoblot and immunofluorescence analyses revealed the up-regulation of PARP1 in remodeled right ventricle with prominent nuclear localization in hypertrophied CMs from decompensated right ventricle patients with PAH. Consistently, a striking increase of poly(adenosine diphosphate–ribose) levels, reflecting PARP1 activity, was observed in CMs from decompensated right ventricle from both humans and rodents (Figures 1A and 2B, Supplemental Figure 1B). Increased expression of PKM2 was observed during RV remodeling, with a more pronounced increase at the decompensated stage, while no significant change was found for the muscle isozyme 1 isoform (Figures 1B and 2C, Supplemental Figure 1C). In support of this, phosphorylation of the Y105 residue of PKM2 (indicative of its dimeric form and nuclear localization^29^) was increased in human and rodent decompensated right ventricle (Figures 1B and 2C, Supplemental Figure 1C). Taken together, these findings indicate that monocrotaline and PAB rat models largely reproduce the histologic events seen in humans and that up-regulation of PARP1 expression and activity along with increased levels of total and phosphorylated forms of PKM2 represents a common denominator of maladaptive RV remodeling in patients with PAH and animal models.

### Inhibition of PARP1 and cytosolic retention of PKM2 prevent myocyte dysfunction under exposure to stress inducers

**Figure 3:**
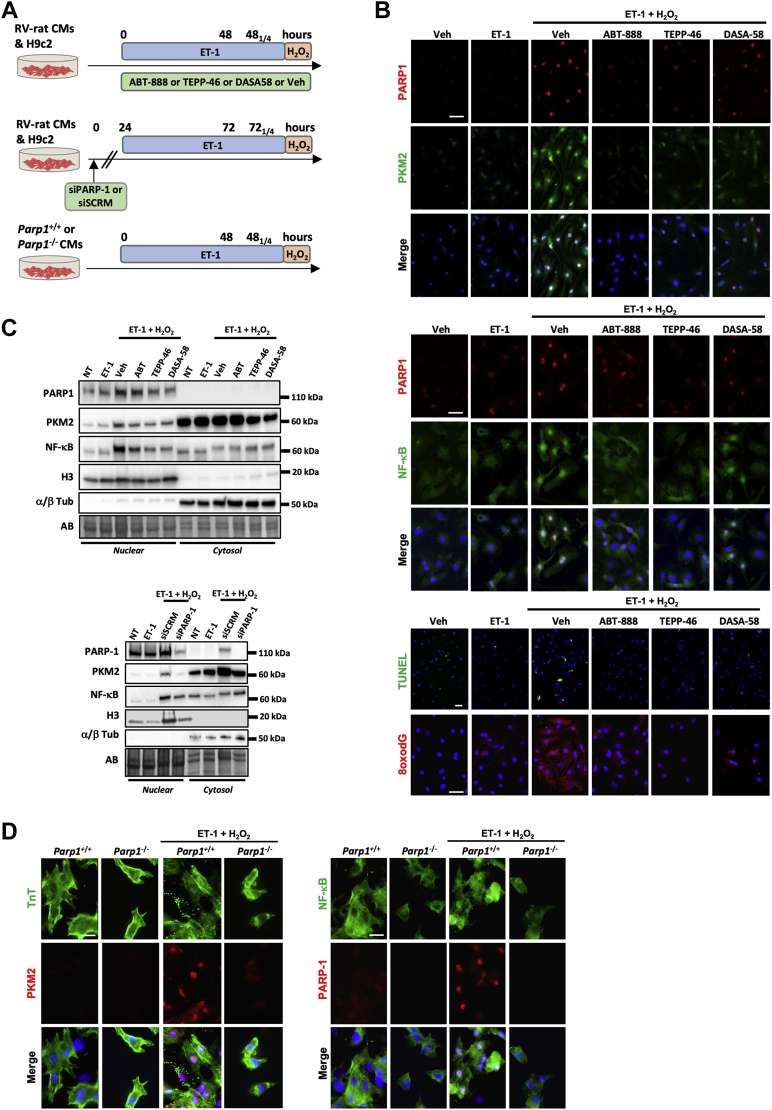
Inhibition of PARP1 and Cytosolic Retention of PKM2 Prevent CM Dysfunction Under Exposure to Oxidative Damage **(A)** Schematic of the experimental design. **(B)** Representative immunofluorescent images of PARP1, PKM2, nuclear factor κB (NF-κB), TUNEL, and cytosolic 8-Oxo-2′-deoxyguanosine (8oxodG) in neonatal rat RV CMs (n = 3 or 4) exposed or not to either endothelin-1 (ET-1) or ET-1 + H_2_O_2_ in the presence or absence of ABT-888, TEPP-46, and DASA-58. **(C)** Representative western blots (of 3 independent studies) for PARP1, PKM2, NF-κB, α/β tubulin (Tub), and histone H3 in nuclear and cytosolic extracts from H9c2 cells exposed or not to either ET-1 or ET-1 + H_2_O_2_ in the presence or absence of ABT-888, TEPP-46, DASA-58, and siPARP1. **(D)** Representative immunofluorescent images of TnT, PKM2, NF-κB, and PARP1 in neonatal CMs isolated from wild-type (*Parp1*^+/+^, n = 3) and *Parp1*-knockout (*Parp1*^−/−^, n = 3) mice exposed or not to ET-1 + H_2_O_2_. Scale bars, 25 mm. NT = non-treated; siSCRM = scrambled siRNA; Veh = vehicle; other abbreviations as in Figure 1.

The transition from adaptive to maladaptive CM remodeling is attributed to the long-term cumulative effect of interconnected stressors.^3^^,^^4^^,^^7^^,^^8^ To model the stressful environment CMs face during maladaptive cardiac remodeling, H9c2 and neonatal rat CMs derived from right ventricle were sequentially exposed to the prohypertrophic factor endothelin-1 (ET-1) for 48 hours before stimulation or not with H_2_O_2_, a DNA damage inducer and PARP1 activator (Figure 3A). In this model, we first analyzed the expression and activity of PARP1 and PKM2. As demonstrated by immunofluorescence, a robust nuclear expression of PARP1 and PKM2 was detected in NCRMs exposed to ET-1 + H_2_O_2_ but not in cells treated with ET-1 alone (Figure 3B, Supplemental Figure 4A). This was accompanied by nuclear translocation of NF-κB, increased cytosolic 8-Oxo-2′-deoxyguanosine intensity (indicative of oxidative DNA damage), and cell death (Figure 3B, Supplemental Figure 4A), thus recapitulating the maladaptive features of the decompensated stage. We next investigated whether pharmacologic inhibition of PARP1 using ABT-888 or enforced cytosolic retention of PKM2 using TEPP-46 or DASA-58 prevents these effects. As shown in Figure 3 and Supplemental Figure 4, diminished immunoreactivity of PARP1 and PKM2 was observed in neonatal rat CMs exposed to ABT-888, TEPP-46, or DASA-58. Furthermore, both PARP1 inhibition and cytosolic PKM2 activation resulted in reduced nuclear translocation of NF-κB, decreased 8-Oxo-2′-deoxyguanosine intensity, and prevention of CM death (Figure 3B, Supplemental Figure 4A). To corroborate these results, cytosolic and nuclear fractions from H9c2 similarly subjected to ET-1 ± H_2_O_2_ exposure were prepared. The purity of both fractions was validated using different fraction-specific protein antibodies (α/β-tubulin for cytosolic compartment and histone H3 for nuclear extract), thus confirming the high levels of separation of each fraction (Figure 3C). As observed by immunofluorescence, exposure of H9c2 to ET-1 + H_2_O_2_ induced nuclear translocation of PKM2 and NF-κB. These effects were suppressed by treatment with ABT-888, TEPP-46, or DASA-58 (Figure 3C). To complement this pharmacologic approach, molecular inhibition of PARP1 was performed using small interfering RNA. Immunofluorescence experiments combined to immunoblots on nuclear and cytosolic extracts showed that knockdown of PARP1 similarly attenuated ET-1 + H_2_O_2_–induced nuclear translocation of PKM2 and NF-κB as well as cell death (Figure 3C, Supplemental Figure 4B). Finally, we used a genetic approach and exposed CMs isolated from wild-type (WT) and *Parp1*-knockout mice to either ET-1 or ET-1 + H_2_O_2_. As observed after pharmacologic or molecular inhibition of PARP1, *Parp1* loss of function resulted in suppressed PKM2 immunoreactivity and NF-κB translocation (Figure 3D, Supplemental Figure 4C).

**Figure 4:**
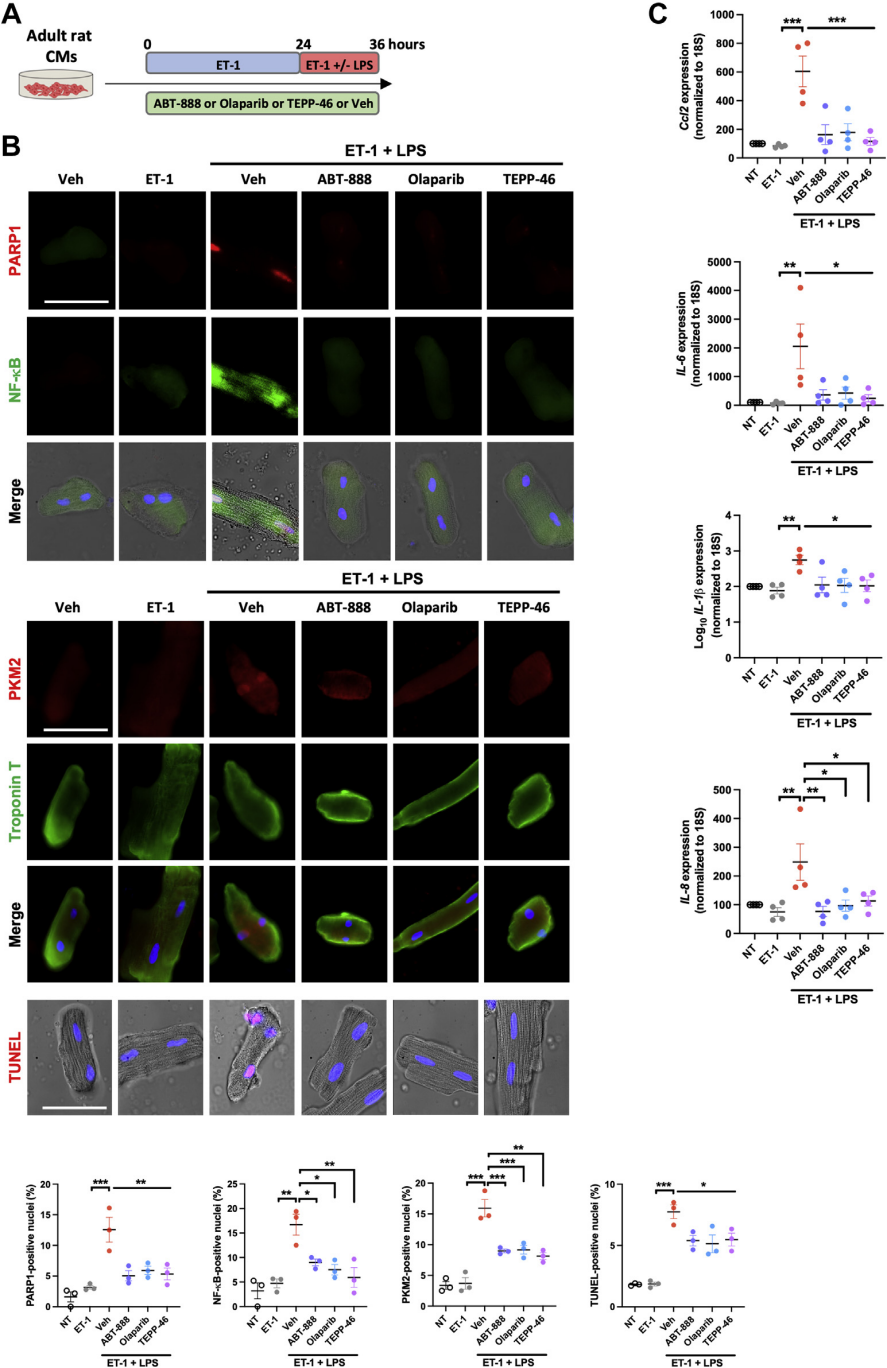
Inhibition of PARP1 and Cytosolic Retention of PKM2 Prevent CM Dysfunction Under Exposure to LPS **(A)** Schematic of the experimental design. **(B)** Representative immunofluorescent images of PARP1, NF-κB, PKM2, TnT, and TUNEL in adult rat CMs (n = 3) exposed or not to either ET-1 or ET-1 + lipopolysaccharide (LPS) in the presence or absence of ABT-888, olaparib, and TEPP-46. Quantification of the percentage of adult rat CMs exhibiting nuclear staining of PARP1, PKM2, NF-κB, and TUNEL is presented. **(C)** Relative messenger RNA levels of *Ccl2*, *IL-1β*, *IL-6*, and *IL-8* in adult rat CMs (n = 4) exposed or not to either ET-1 or ET-1 + LPS in the presence or absence of ABT-888, olaparib, and TEPP-46. Scale bars, 50 mm. ∗*P <* 0.05, ∗∗*P <* 0.01, and ∗∗∗*P <* 0.001. Scatter dot plots show individual values and mean ± SEM. Comparisons were made using 1-way analysis of variance followed by Tukey multiple-comparison tests. Abbreviations as in Figures 1 and 3.

Because the factors triggering and sustaining CM dysfunction are multiple and mutually reinforce one another, a second experimental setup was established allowing examination of the ability of PARP1/PKM2 modulators to prevent inflammatory-mediated adult CM dysfunction. CMs isolated from adult rats were treated with ET-1 for 24 hours and then coexposed with ET-1 and lipopolysaccharide for 12 hours in the presence or not of PARP1 inhibitors or PKM2 activators (Figure 4A). As observed upon treatment with ET-1 + H_2_O_2_, ET-1 + lipopolysaccharide exposure led to increased nuclear expression of PARP1, PKM2, and NF-κB, accompanied by a higher proportion of cells positive on terminal deoxynucleotidyl transferase dUTP nick-end labeling assay (Figure 4B). These effects were prevented by ABT-888, olaparib, or TEPP-46 (Figure 4B). Similar results were obtained in H9c2 (Supplemental Figures 5A and 5B). Furthermore, both PARP1 inhibition or PKM2 activation attenuated ET-1 + lipopolysaccharide–induced expression of proinflammatory (pSTAT3, *IL-6*, *IL-8*, *Ccl2*, and *Socs3*) and glycolytic (lactate dehydrogenase, hexokinase 2, and cMYC) markers (Figure 4C, Supplemental Figures 5C and 6). Taken together, these data demonstrate a robust connection between overactivation of PARP1 and dimeric PKM2 in CM dysfunction.

### Inhibition of PARP1 prevents cardiac fibroblast activation

**Figure 5:**
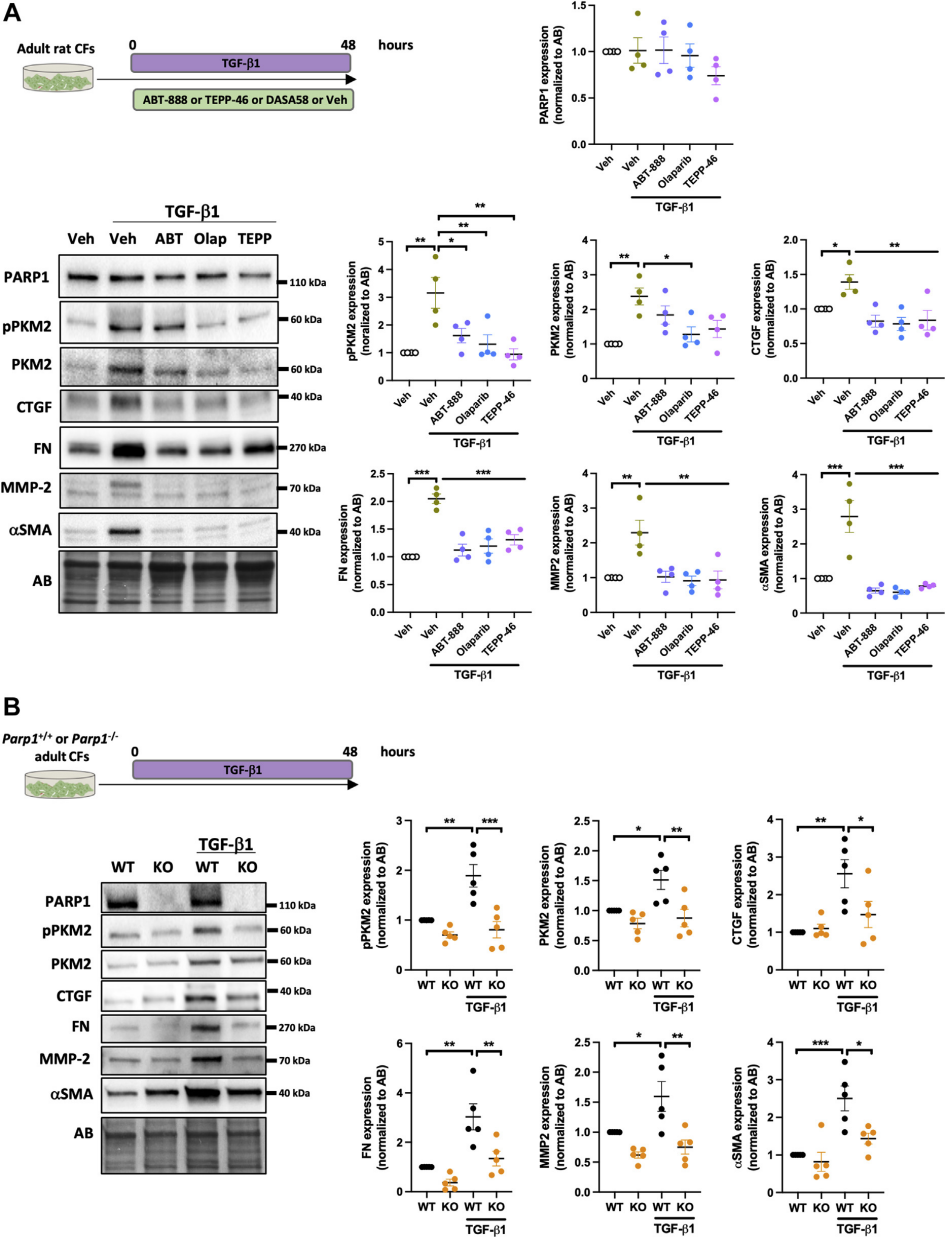
Inhibition of PARP1 and Cytosolic Retention of PKM2 Attenuate Cardiac Fibroblast Activation **(A)** Schematic of the experimental design, representative western blots, and quantification of PARP1, pPKM2, PKM2, connective tissue growth factor (CTGF), fibronectin (FN), matrix metalloproteinase–2 (MMP-2) and alpha smooth muscle actin (αSMA) in adult rat cardiac fibroblasts (CFs; n = 4) exposed or not to transforming growth factor–β1 (TGF-β1) (5 ng/mL) in the presence or absence of ABT-888, olaparib (Olap), or TEPP-46. **(B)** Schematic of the experimental design, representative western blots, and quantification of PARP1, pPKM2, PKM2, CTGF, FN, MMP-2, and αSMA in adult CFs isolated from wild-type (WT; n = 5) of *Parp1*-knockout (KO; n = 5) mice treated or not with TGF-β1 (5 ng/mL). ∗*P <* 0.05, ∗∗*P <* 0.01, ∗∗∗*P <* 0.001. Scatter dot plots show individual values and mean ± SEM. Comparisons were made using 1-way analysis of variance followed by Tukey multiple-comparison tests. Abbreviations as in Figures 1 and 3.

Although the overexpression of PARP1 was obviously detected in human and rodent CMs from decompensated right ventricle, its implication in cardiac fibroblasts (main effector of fibrosis and tissue stiffness) cannot be ruled out. To gain insight into the role of PARP1/PKM2 in cardiac fibroblast activation, adult rat cardiac fibroblasts were exposed or not to transforming growth factor–β1 for 48 hours (Figure 5A). As expected, treatment with transforming growth factor–β1 resulted in a significant increase in alpha smooth muscle actin, fibronectin, connective tissue growth factor, and matrix metalloproteinase–2, all well-established markers of fibroblast activation (Figure 5A). These effects were accompanied by increased expression of the total and phosphorylated forms of PKM2. We found that inhibition of PARP1 or cytosolic retention of PKM2 similarly blocked the ability of transforming growth factor–β1 to induce the expression of these key fibrotic markers (Figure 5A). To consolidate our findings, a similar approach was conducted in cardiac fibroblasts isolated from WT and *Parp1*^−/−^ mice. As anticipated, genetic inactivation of *Parp1* inhibited transforming growth factor–β1–mediated fibrotic response in mouse cardiac fibroblasts along with PKM2 expression (Figure 5B).

### Pharmacologic inhibition of PARP1 and enforced tetramerization of PKM2 prevent RV dysfunction in PAB-subjected rats

**Figure 6:**
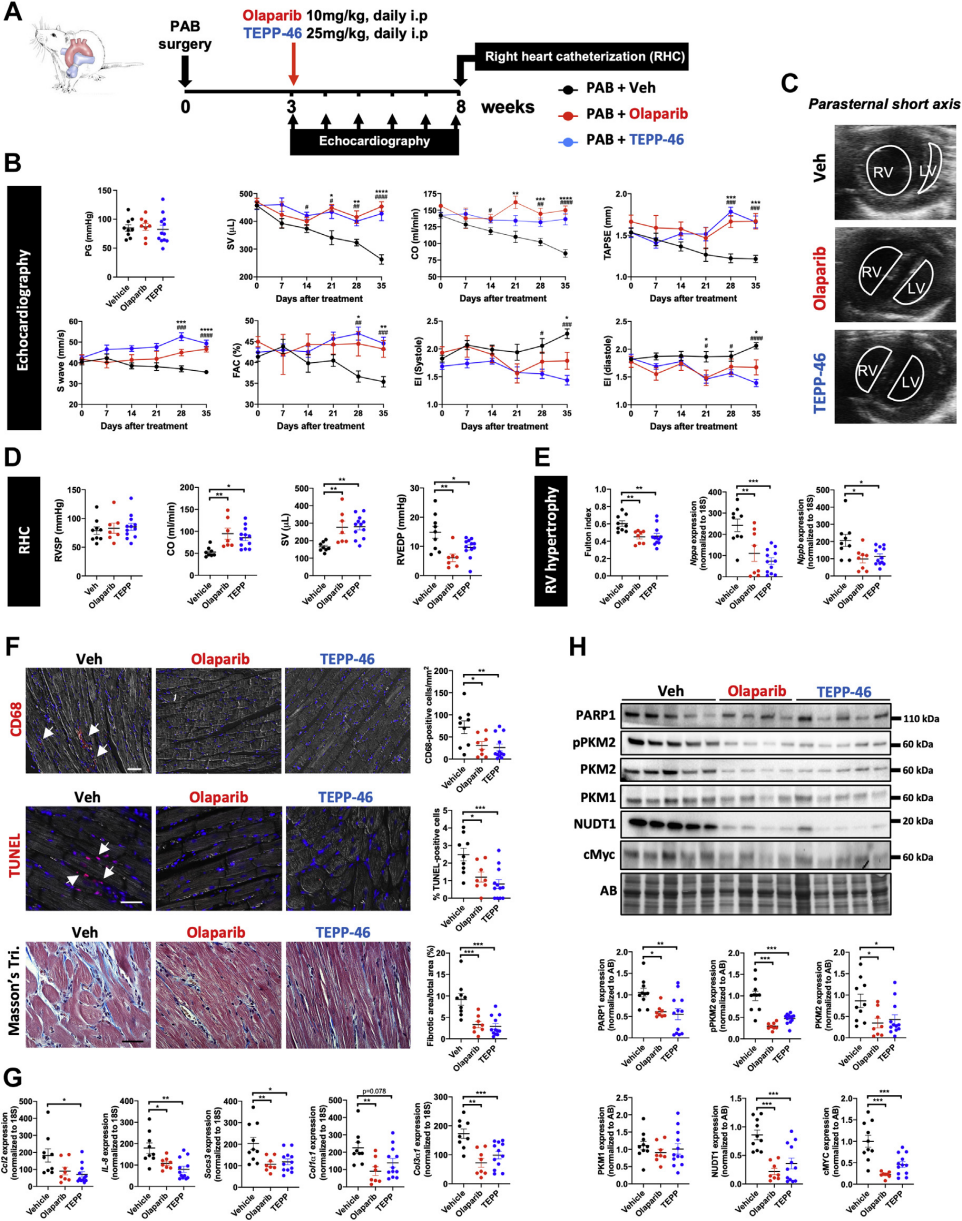
Cardioprotective Effects of PARP1 and PKM2 Modulators in Rats Subjected to PAB **(A)** Schematic representation of the experimental protocol for induction and therapeutic intervention in a PAB-induced RV dysfunction model in rats. **(B)** Pressure gradient (PG) at PAB site, SV, CO, tricuspid annular plane systolic excursion (TAPSE), S′ wave, RV fractional area change (FAC), and systolic and diastolic eccentricity index (EI) measured using echocardiography in PAB rats before and every week after treatment or not with olaparib or TEPP-46. **(C)** Representative echocardiographic images of the right and left ventricles obtained from a parasternal short-axis view in PAB rats treated with olaparib, TEPP-46, or Veh at 5 weeks after the initiation of the treatment. **(D)** RV systolic pressure (RVSP), CO, SV, and RV end-diastolic pressure (RVEDP) measured using right heart catheterization (RHC) at the end of the protocol in PAB rats treated or not with olaparib or TEPP-46. **(E)** RV hypertrophy by Fulton index and relative messenger RNA (mRNA) levels of *Nppa* and *Nppb* expression in PAB rats treated or not with olaparib or TEPP-46. **(F)** Representative images and corresponding quantifications of CD68, TUNEL, and Masson’s trichrome (Tri.) staining in RV sections from PAB rats treated or not with olaparib or TEPP-46. **(G)** Relative mRNA levels of *Ccl2*, *IL-8*, *Socs3*, *Col1α1*, and *Col3α1* in PAB rats treated or not with olaparib or TEPP-46. **(H)** Representative western blots and quantification of PARP1, pPKM2, PKM2, PKM1, Nudix hydrolase 1 (NUDT1), and cMYC in right ventricles from PAB rats exposed or not to olaparib or TEPP-46. Scale bars, 25 mm. **Arrows** denote positive cells. n = 7 to 12 per group. ∗*P <* 0.05, ∗∗*P <* 0.01, and ∗∗∗*P <* 0.001. Scatter dot plots show individual values and mean ± SEM. Comparisons were made using 1-way analysis of variance followed by Tukey multiple-comparison tests or nonparametric Kruskal-Wallis tests. i.p = intraperitoneal; LV = left ventricle; other abbreviations as in Figures 1 and 2.

On the basis of our in vitro findings supporting PARP1 activation and nuclear PKM2 as interconnected drivers of cardiac cell dysfunction, we next evaluated the therapeutic potential of PARP1 inhibitor and PKM2 activator in rats subjected to RV pressure overload induced by PAB. Three weeks after surgery, once concentric hypertrophy was confirmed by echocardiography, rats were randomly assigned to the following 3 study groups: 1) olaparib 10 mg/kg intraperitoneal daily; 2) TEPP-46 25 mg/kg intraperitoneal daily; and 3) vehicle. RV structure and function were noninvasively monitored every week, from weeks 3 to 8 post-PAB (Figure 6A). No significant differences in constriction degree and RV systolic pressure were seen among the PAB groups (Figure 6B). As expected, longitudinal assessments revealed a worsening of RV dysfunction in vehicle-treated PAB rats, as illustrated by progressive declines in stroke volume, CO, tricuspid annular plane systolic excursion, S′ wave, and RV fractional area change (Figure 6B, Supplemental Figure 7A). In contrast, values of CO, stroke volume, RV fractional area change, tricuspid annular plane systolic excursion, and S′ wave were maintained in PAB rats treated with either olaparib or TEPP-46 (Figure 6B, Supplemental Figure 7A). In concert with these changes, the leftward systolic and diastolic interventricular septal displacement measured by the eccentricity index was lower in olaparib- and TEPP-46–treated rats (Figures 6B and 6C). At the end of the treatment period, these noninvasive results were confirmed by right heart catheterization. Indeed, CO and stroke volume were significantly increased, whereas RV end-diastolic pressure was decreased in olaparib- or TEPP-46–treated PAB rats compared with PAB rats receiving vehicle (Figure 6D). No change in RV systolic pressure was noted between olaparib- or TEPP-46–treated and vehicle-treated animals (Figure 6D). RV hypertrophy, calculated using the Fulton index, was diminished upon PARP1 inhibition or PKM2 activation (Figure 6E). Consistent with these findings, the mean RV CM surface area and expression levels of cardiac stress markers (ie, *Nppa*, *Nppb*, and *Myh7*/*Myh6* ratio) were reduced in olaparib- and TEPP-46–treated rats (Figure 6E, Supplemental Figure 7C). As demonstrated by Masson’s trichrome staining and assessment of collagen type 1 alpha 1 chain (*Col1α1*) and collagen type 3 alpha 1 chain (*Col3α1*) expression, the extent of perivascular and interstitial fibrosis was diminished after treatment with olaparib or TEPP-46 (Figures 6F and 6G). Furthermore, inhibition of PARP1 or enforced tetramerization of PKM2 significantly prevented the infiltration of CD4/CD8-positive T cells and cluster of differentiation 68–positive macrophages (Figure 6F, Supplemental Figure 7). Accordingly, lower transcript and/or protein levels of proinflammatory factors (IL-6, IL-1β, IL-8, CCL2, and SOCS3) were observed (Figure 6G, Supplemental Figure 7). Compared with vehicle-treated PAB rats, marked decreases of PARP1, PKM2, pPKM2, and pSTAT3 as well as cMYC, lactate dehydrogenase, and hexokinase 2 were detected in both olaparib- and TEPP-46–treated groups, supporting the fact that improvement of RV function resulted from the reversal of the glycolytic shift (Figure 6H, Supplemental Figure 8). Finally, the administration of olaparib or TEPP-46 markedly lowered the expression of Nudix hydrolase 1 (Figure 6H), a marker of oxidative DNA damage, and attenuated the percentage of terminal deoxynucleotidyl transferase dUTP nick-end labeling–positive CMs (Figure 6F). Altogether, these data clearly demonstrate that olaparib and TEPP-46 exert direct cardioprotective effects on the right ventricle.

### *Parp1* gene inactivation confers protection against PAB-induced RV dysfunction

**Figure 7:**
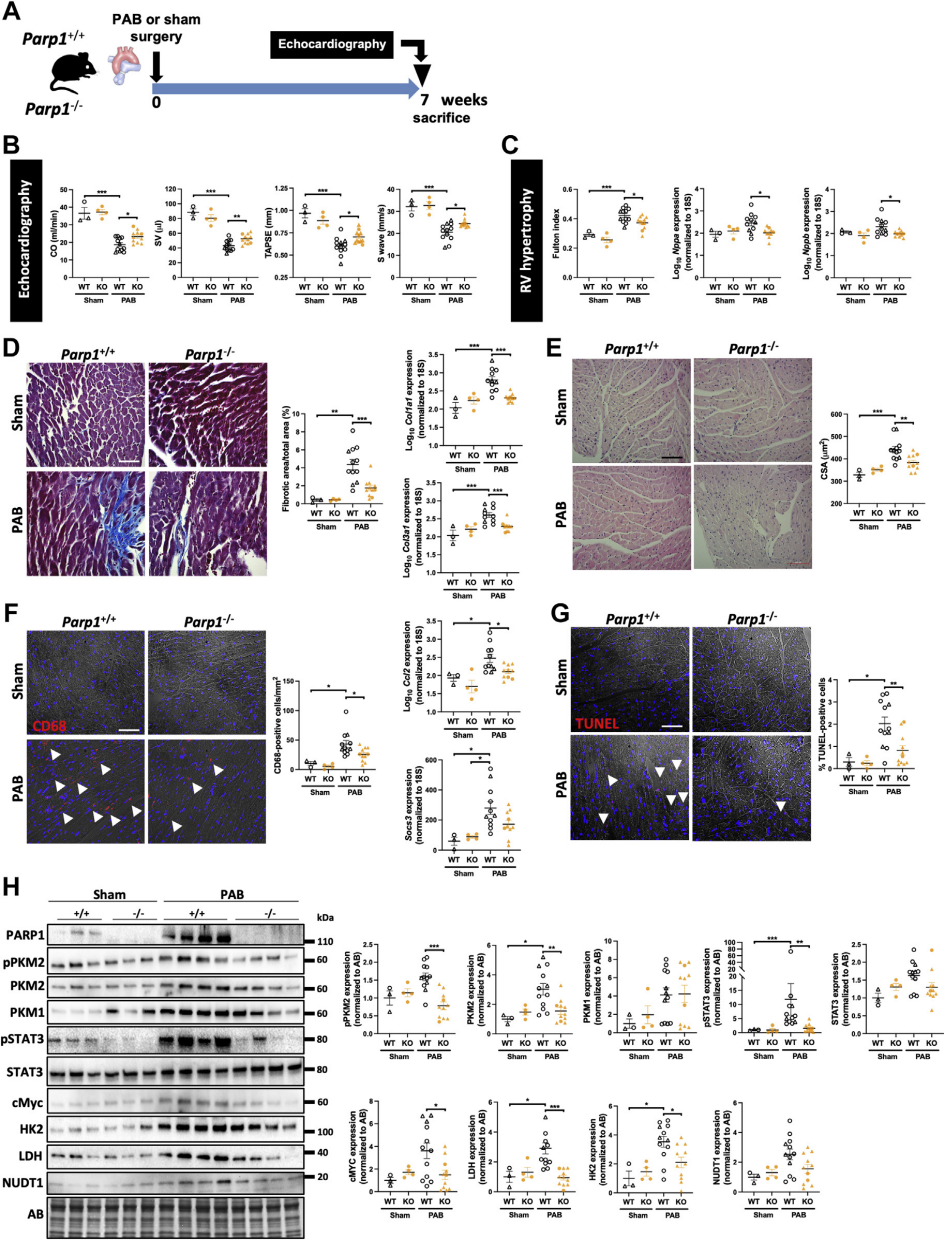
Parp1 Loss of Function Confers Protection Against PAB-Induced RV Dysfunction **(A)** Schematic of the experimental design. **(B)** CO, SV, TAPSE, and S′ wave measured using echocardiography in WT and *Parp1*-KO mice 7 weeks after PAB or sham surgery. **(C)** RV hypertrophy by Fulton index and relative mRNA levels of *Nppa* and *Nppb* expression in mice. **(D)** Representative images and quantification of Masson’s trichrome staining and relative mRNA levels of *Col1α1* and *Col3α1* expression in right ventricles from WT and *Parp1*-KO mice subjected to PAB or sham surgery. **(E)** Representative images of hematoxylin and eosin–stained RV sections and quantification of CSA. **(F)** Representative images and quantification of infiltrated macrophages stained with anti-CD68. Relative mRNA levels of *Ccl2* and *Socs3* in right ventricles of 7-week sham- or PAB-operated WT and *Parp1*-KO mice. **(G)** Representative images of TUNEL-stained RV sections and quantification of the percentage of positive cells. **(H)** Representative western blots and quantification of PARP1, pPKM2, PKM2, PKM1, phosphorylated signal transducer activator of transcription 3 (pSTAT3), STAT3, cMYC, hexokinase 2 (HK2), lactate dehydrogenase (LDH), and NUDT1 in right ventricles from WT and *Parp1*-KO mice subjected to PAB or sham surgery. Scale bars, 50 mm. **Arrowheads**, positive cells. WT sham, n = 3; *Parp1*-KO sham, n = 4; WT PAB, n = 11; and *Parp1*-KO, n = 10. Female mice are indicated by **triangular symbols**, and male mice are indicated by **circular symbols**. ∗*P <* 0.05, ∗∗*P <* 0.01, and ∗∗∗*P <* 0.001. Scatter dot plots show individual values and mean ± SEM. Comparisons were made using 1-way analysis of variance followed by Tukey multiple-comparison tests or nonparametric Kruskal-Wallis tests. Abbreviations as in Figures 1, 2, 5, and 6.

To further confirm PARP1 inhibition as a therapy to support RV function, a genetic approach was carried out. Adult male and female WT and *Parp1*-knockout mice were subjected to PAB (Figure 7A). A group of sham-operated WT and mutant mice served as controls. We found that sham-operated *Parp1*^−/−^ mice display no overt RV phenotype at baseline (Figure 7B). After 7 weeks post-PAB procedures, echocardiography showed significant reductions in stroke volume, CO, tricuspid annular plane systolic excursion, and S′ wave in *Parp1*^+/+^ mice compared with the sham groups, confirming the features of RV dysfunction (Figure 7B). In contrast, these effects were attenuated in *Parp1*^−/−^ mice (Figure 7B). Moreover, the marked increase in Fulton index, CM surface area, fibrotic area, messenger RNA levels of *Nppa*, *Nppb*, *Col1α1*, and *Col3α1* observed in *Parp1*^+/+^ PAB mice were significantly reduced in *Parp1*^−/−^ mice (Figures 7C and 7D). PAB-operated *Parp1*^+/+^ mice also exhibited more RV infiltration of T cells and monocytes and macrophages and higher messenger RNA expression of Ccl2, Socs3, IL-1β, and IL-6 than PAB-operated *Parp1*^−/−^ mice (Figure 7F, Supplemental Figure 9). Additionally, PAB-induced CM apoptosis was alleviated by loss of *Parp1* function (Figure 7G). Consistent with our in vitro and in vivo observations, the expression levels of PARP1, phosphorylated PKM2, total PKM2, pSTAT3, cMYC, hexokinase 2, lactate dehydrogenase, and Nudix hydrolase 1 were markedly increased in WT animals subjected to PAB, whereas these effects were significantly blunted in mutant PAB mice, except for Nudix hydrolase 1, which exhibited a decreasing trend (Figure 7H). No significant changes in pyruvate kinase muscle isozyme 1 protein levels were noticed in the right ventricle from *Parp1*^+/+^ and *Parp1*^−/−^ mice that underwent PAB (Figure 7H). These results provide additional compelling evidence in support to a critical role of PARP1 in mediating maladaptive RV remodeling.

### CM-specific *Parp1* deletion improves RV function in mice subjected to Sugen/hypoxia

**Figure 8:**
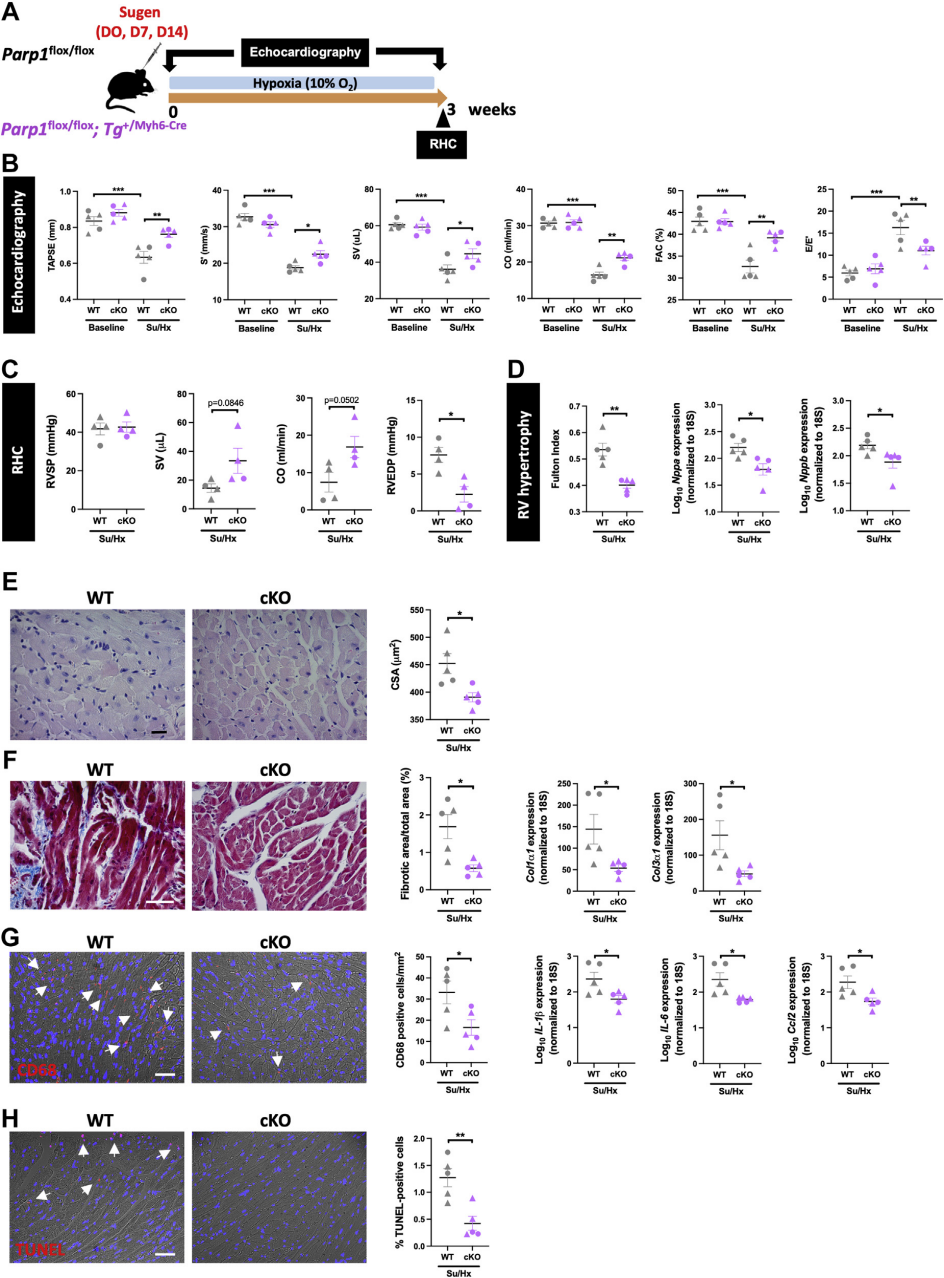
CM-Targeted Parp1 Inactivation Improves RV Function in Mice Exposed to Su/Hx **(A)** Schematic of the experimental design. **(B)** TAPSE, S′ wave, SV, CO, RV FAC, and RV E/E′ ratio measured using echocardiography in *Parp1*^flox/flox^ (WT) and *Parp1*^flox/flox^;*Tg*^+/Myh6-Cre^ (conditional KO [cKO]) mice at baseline and after exposure to Sugen/hypoxia (Su/Hx). **(C)** RVSP, SV, CO, and RVEDP measured using RHC at the end of the protocol in Su/Hx-exposed WT and conditional mutant mice. **(D)** RV hypertrophy by Fulton index and relative mRNA levels of *Nppa* and *Nppb* expression in mice. **(E)** Representative images of hematoxylin and eosin–stained RV sections and quantification of CM surface area. **(F)** Representative images and quantification of Masson’s trichrome staining and relative mRNA levels of *Col1α1* and *Col3α1* expression in right ventricles from Su/Hx-treated WT and cKO mice. **(G)** Representative images and quantification of infiltrated macrophages stained with anti-CD68. Relative mRNA levels of *IL-1β*, *IL-6*, and *Ccl2* in right ventricles of mice. **(H)** Representative images of TUNEL-stained RV sections and quantification of the percentage of positive cells. Scale bars, 50 mm. **Arrowheads** denote positive cells. n = 4 or 5 per group. Female mice are indicated by **triangular symbols**, and male mice are indicated by **circular symbols**. ∗*P <* 0.05, ∗∗*P <* 0.01, and ∗∗∗*P <* 0.001. Scatter dot plots show individual values and mean ± SEM. Comparisons were made using 1-way analysis of variance followed by Tukey multiple-comparison tests and Student’s *t-test* or the Mann-Whitney *U* test. Abbreviations as in Figures 1 and 6.

To determine whether PARP1 overactivity in CMs plays a significant role in RV failure, CM-specific *Parp1* gene loss of function was achieved by crossing *Parp1*^flox/flox^ mice with mice harboring Myh6-driven Cre recombinase. Adult mutant (*Parp1*^flox/flox^;*Tg*^+/Myh6-Cre^, hereafter referred as conditional knockout [cKO]) and control (*Parp1*^flox/flox^ designated as WT) mice were next exposed to Sugen/hypoxia to induce pulmonary hypertension. Echocardiography was performed at baseline and 3 weeks after Sugen/hypoxia exposure in both cKO and WT mice (Figure 8A). Before Sugen/hypoxia intervention, assessment of RV function revealed no significant difference between WT and cKO mice (Figure 8B). As expected, RV dysfunction was evident in WT mice challenged with Sugen/hypoxia for 3 weeks, as reflected by marked decreases in tricuspid annular plane systolic excursion, CO, cardiac index, stroke volume, S′ wave, and fractional area change (Figure 8B). Consistently, CO and stroke volume obtained by right heart catheterization were found to be augmented and RV end-diastolic pressure decreased in Sugen/hypoxia–exposed cKO mice, whereas no changes in RV systolic pressure and pulmonary vascular remodeling were seen (Figure 8C, Supplemental Figure 10A). These RV function improvements were accompanied by a reduced Fulton index (Figure 8D) associated with lower CM surface area (Figure 8E). The RV molecular signature was also indicative of ameliorated RV remodeling in cKO mice, with lower expression of *Nppa* and *Nppb* as well as *Myh7*/*Myh6* ratio (Figure 8D, Supplemental Figure 10B). RV fibrosis was more severe in WT Sugen/hypoxia mice compared with cKO Sugen/hypoxia mice (Figure 8F). In agreement with this, the expression levels of *Col1α1* and *Col3α1* were significantly reduced in CM-restricted *Parp1*-deficient mice (Figure 8F). Finally, the infiltration of T cells and macrophages and the expression of proinflammatory factors, including *IL-6*, *IL-1β*, *Ccl2*, and *Socs3*, were substantially diminished in Sugen/hypoxia cKO mice (Figure 8G, Supplemental Figure 10C), denoting the CM-specific deleterious role of PARP1 overactivation in maladaptive RV remodeling. The beneficial effects of CM-restricted *Parp1* inactivation on RV structure and function were associated with down-regulation of pPKM2, PKM2, hexokinase 2, lactate dehydrogenase, cMYC, pSTAT3, and Nudix hydrolase 1 expression levels (Supplemental Figure 11).

## Discussion

Numerous preclinical studies have been undertaken to show the beneficial effects of many compounds for reversing pulmonary vascular remodeling in PAH. However, direct evidence of their safety or even their cardioprotective effects has rarely been researched and even less commonly documented. This is even more important given that pulmonary antiremodeling approaches seek to halt proliferation and induce apoptosis, whereas loss of CMs and associated fibrosis are considered as cardinal features of maladaptive RV remodeling. Although inhibition of the multifaceted proteins PARP1 and PKM2 have been demonstrated to elicit therapeutic effects in preclinical models in reversing pulmonary vascular remodeling,^13^^,^^24^ their safety or even direct cardioprotective effects in the setting of PAH have never been investigated.

In the present study, we showed that PARP1 activity and nuclear PKM2 localization are specifically increased during maladaptive RV remodeling. By using complementary in vitro loss-of-function approaches, we found that overactivated PARP1 promotes CM dysfunction by favoring PKM2 expression and nuclear function, glycolytic gene expression, nuclear translocation of NF-κB, and increased expression of proinflammatory factors. More important, we found that both pharmacologic and genetic inhibition of PARP1 along with enforced cytosolic retention of PKM2 significantly attenuate RV dysfunction in animals subjected to PAB, a model allowing interrogation of RV responses independent of pulmonary vascular effects. Although direct extrapolation of knowledge gained from left heart failure experiments to the right side is considered as uncertain,^4^^,^^8^ our findings are nevertheless consistent with published data showing that the inhibition of PARP1 exerts cardioprotective effects in various animal models of left heart failure.^30^, ^31^, ^32^, ^33^ Mounting evidence supports the view that the up-regulation of PARP1 represents a double-edged sword, with mild activation of PARP1 facilitating DNA repair and cell survival while its prolonged overactivation has adverse effects, ultimately causing cell energy crisis and cell death through depletion of intracellular nicotinamide adenine dinucleotide^+^/adenosine triphosphate pool.^34^ Consistently, *Parp1* gene inactivation and nicotinamide adenine dinucleotide replenishment were reported to protect CMs from angiotensin II–induced cell death.^31^ In addition to provoking cell death, which triggers inflammatory reactions by multiple mechanisms, extensive and sustained activation of PARP1 in cancer cells was documented to promote glycolysis and exacerbate inflammation,^23^^,^^35^^,^^36^ 2 key components in the initiation and progression of RV failure.^5^^,^^37^ Indeed, a large body of research has demonstrated that a complex crosstalk exists among PARP1, PKM2, NF-κB, cMYC, and STAT3 that nourishes a vicious circle. For instance, PARP1 was reported to stimulate PKM2 expression via STAT3 and NF-κB, contributing to the Warburg effect and inflammation.^38^ Similarly, PARP1-dependent PARylation of PKM2 was demonstrated to promote its nuclear retention.^23^ In turn, PKM2 was shown to phosphorylate STAT3, contributing to its proinflammatory function.^21^ Additionally, a positive feedback loop among cMYC, PKM2, and PARP1 has been described, with cMyc favoring the heterogeneous nuclear ribonucleoprotein–dependent regulation of PKM2 gene splicing^39^^,^^40^ as well as PARP1 expression and activity.^41^ Finally, nuclear PKM2 is required for the expression of cMYC^42^ and NF-κB P65 nuclear retention, contributing to abnormal energy metabolism and continuous activation of inflammatory signaling pathways.^43^ In support of this model pinpointing PARP1 and PKM2 as integral pieces of a self-perpetuating metabolic-inflammatory vicious circle of tissue damage, we found that blocking PARP1 activity or nuclear PKM2 translocation reduces PARP1 and PKM2 abundance in cells and tissues. Accordingly, the beneficial effects of PARP1 inhibition on RV function in PAB animals were largely recapitulated by PKM2 cytosolic activation.

Interestingly, we also provided evidence that the deleterious effects of PARP1 overactivation during RV remodeling are not restricted to CMs, as its inhibition markedly represses transforming growth factor–β1–induced differentiation of cardiac fibroblasts in vitro and reduced RV fibrosis in vivo. The cell-autonomous antifibrotic role of PARP1 in cardiac fibroblasts is solidified by several studies showing that inhibition of PARP1 activates lung myofibroblast activation, whereas pharmacologic or molecular suppression of its activity exerts opposite functions.^44^, ^45^, ^46^ Nevertheless, or findings showing that CM-restricted inactivation of *Parp1* confers protection against Sugen/hypoxia–induced RV dysfunction suggest that PARP1 overexpression in CMs is a major determinant of RV failure.

The pathophysiology underlying RV failure is complex and multifactorial, and as a consequence, in vitro models that recapitulate the adaptive and maladaptive features of CMs are imperfect. Nevertheless, the beneficial effects of PARP1/PKM2 modulators observed in our 2 complementary in vitro models combining rat cardiomyoblasts and CMs coupled with our in vivo results clearly support the therapeutic potential of both in RV failure. As inhibition of PARP1 using olaparib is currently tested in patients with PAH (OPTION [Olaparib for PAH: a Multicenter Clinical Trial]; NCT03782818), the long-term efficacy, safety, and tolerability of PARP1 inhibition in preclinical models was not assessed.

### Study limitations

Although our human RV biobank has been shown in the past to be very efficient to study RV dysfunction,^26^^,^^47^^,^^48^ as always, the use of human tissues has certain limitations. First, control samples are not complete healthy, although they come from patients who had diseases known to not affect RV function, as indicated by hemodynamic measurements. Compared with decompensated right ventricles, compensated right ventricles are from younger adult patients who underwent corrective surgery for congenital heart disease (mostly pulmonary valve stenosis), while decompensated right ventricles are from patients with end-stage PAH. Nonetheless, the RV molecular, cellular, and histologic pattern seen in these patients is similar to what is seen in animal models. Therefore, despite inherent limitations of each animal model, the fact that a similar pattern was seen in all of them and the fact that a combination of models was used greatly minimize these limitations and in all aspects accord with preclinical research guidelines in the field.^25^^,^^49^

## Conclusions

We found that that interference with PARP1-PKM2 signaling delivers a combined attack on multiple cell types and multiple detrimental mechanisms underlying pathological RV remodeling in addition to reverse pulmonary artery wall thickening.

Perspectives **COMPETENCY IN MEDICAL KNOWLEDGE:** Although the pulmonary vasculature is the locus of the initial insult, short- and long-term outcomes of patients with PAH are dependent largely on their RV function. Despite this, the molecular players engaged in these remodeling events remain only partially understood, contributing to a lack of effective RV-targeted therapies. The present study identified the PARP1/PKM2 axis as a molecular integrator and therapeutic target of oxidative stress, metabolic dysfunction, and tissue inflammation in RV failure. Indeed, pharmacologic and genetic inhibition of PARP1 as well as enforced tetramerization of PKM2 significantly attenuated the typical hallmarks of maladaptive remodeling and directly improved RV function in relevant preclinical animal models.

**TRANSLATIONAL OUTLOOK:** Combined with published data, this study established PARP1/PKM2 axis as a molecular pathway similarly deranged in the RV and pulmonary vasculature, offering the possibility of therapies that simultaneously treat the RV and pulmonary circulation. These results support clinical investigation of Olaparib (OPTION; NCT03782818) as a promising therapy for patients with PAH.

## Funding Support and Author Disclosures

This research was supported by a grant from the Cardiovascular Medical Research and Education Fund to Drs Boucherat and Provencher. Dr Boucherat has been funded by the Canadian Institute of Health Research (IC121617). Dr Boucherat holds a junior scholar award from Fonds de Recherche du Québec: Santé. Dr Bonnet holds a distinguished research scholar from Fonds de Recherche du Québec: Santé. All other authors have reported that they have no relationships relevant to the contents of this paper to disclose.
