# Supplementary material for: PARP1-PKM2 Axis Mediates Right Ventricular Failure Associated With Pulmonary Arterial Hypertension
Source: JACC Basic Transl Sci. 2022 Mar 16;7(4):384–403. doi: 10.1016/j.jacbts.2022.01.005 (PMC9079853; doi:10.1016/j.jacbts.2022.01.005)
Supplement: Supplemental Data [file mmc1.pdf]

Supplemental Appendix

To accompany manuscript:

"PARP1-PKM2 Axis Mediates Right Ventricular Failure Associated with Pulmonary Arterial Hypertension" by Shimauchi et al.

(Short title: PARP1-PKM2 Axis in RV Failure)

Supplemental Material and Methods 2-8

Supplemental Table 1. Clinical information of human RV samples. 10-12
Supplemental Table 2. Summary of human RV samples. 13-14
Supplemental Table 3. Categorization of rats in the monocrotaline (MCT) and pulmonary artery banding (PAB) models of RV failure. 15
Supplemental Table 4. Primer sequences or references used for qPCR. 16-17
Supplemental Table 5. Primary antibodies used for WB and histological analysis. 18

Supplemental Figure 1. Increased expression and activity of PARP1 and PKM2 in decompensated RV from MCT rats. 20-21
Supplemental Figure 2. Increased expression of glycolysis and oxidative DNA damage related proteins in decompensated RV from Human, MCT rats, and PAB rats. 22-23
Supplemental Figure 3. Increased expression of inflammation related genes in decompensated RV from Human, MCT rats, and PAB rats. 24
Supplemental Figure 4. Inhibition of PARP1 as well as cytosolic retention of PKM2 prevent cardiomyocyte dysfunction. 25-26
Supplemental Figure 5. Inhibition of PARP1 and cytosolic retention of PKM2 prevent H9c2 cells dysfunction under exposure to LPS. 27-28
Supplemental Figure 6. Inhibition of PARP1 and cytosolic retention of PKM2 prevent increase of glycolysis and DNA damage related proteins under exposure to LPS and ET-1. 29
Supplemental Figure 7. Cardioprotective effects of PARP1 inhibitor and PKM2 activator in rats subjected to PAB. 30-31
Supplemental Figure 8. Cardioprotective effects of PARP1 inhibitor and PKM2 activator in rats subjected to PAB. 32
Supplemental Figure 9. *Parp1* inactivation attenuates PAB-induced RV inflammation. 33
Supplemental Figure 10. Cardiac specific *Parp1* deletion prevents Su/Hx-induced RV dysfunction regardless of pulmonary vascular remodeling. 34
Supplemental Figure 11. Cardiac specific *Parp1* deletion prevents Su/Hx-induced increase of glycolysis and DNA damage related proteins in RVs. 35

Supplemental References 36

Supplemental Material and Methods

Animal Studies

Male Sprague-Dawley rats (250-300g body weight) were purchased from Charles River. Adult mice with homozygous deletion of *Parp1* (129S-*Parp1*^{tm1Zqw}/J, referred to henceforth as *Parp1*$^{-/-}$)(1) and the corresponding 129S wild-type (WT) strain were purchased from The Jackson Laboratories. To obtain mice with cardiomyocyte-specific *Parp1* deletion (cKO), *Parp1*$^{flox/flox}$ mice (B6(Cg)-*Parp1*$^{tm1c(EUCOMM)/Hmgu}$/WlkrJ)(2) were interbred with mice expressing Cre recombinase linked to murine alpha myosin-heavy chain (*Myh6*) promoter (3), both purchased from The Jackson Laboratories. Littermates *Parp1*$^{flox/flox}$ mice were used as controls.

Rats and mice were group-housed on a standard 12h:12h light:dark cycle and kept in a temperature and humidity-controlled room, with free access to food and water. To reduce the number of animals used to a minimum and thus follow the guiding principles for more ethical use of animals in testing, RV tissues from MCT-injected and PAB-operated rats categorized as compensated or decompensated based on hemodynamic parameters at the time of sacrifice and used in our previous publication (4) were de novo examined (Supplemental Table 3).

Pulmonary Artery Banding (PAB) Animal Models

Adult male Sprague-Dawley rats or male and female WT and *Parp1*$^{-/-}$ mice (12- to 18-weeks old) were randomly subjected to PAB or sham surgery. Briefly, rats and mice were anesthetized with isoflurane and buprenorphine and then intubated. Following local anesthesia with lidocaine, we performed median sternotomy and the PA was dissected free from the aorta and left atrium. The main PA was banded with silk sutures (6-0), tied tight against a 19 (for rats) or 26 (for mice)-gauge needle, which was removed quickly. Sham operated mice underwent the same procedure but without tying the pulmonary trunk. As assessed by echocardiography, PAB-operated rats and mice with peak velocity above 3.5 m/s or 2.0 m/s at banding site, respectively, were enrolled for this study. Three weeks post-PAB, rats were randomly allocated to one the following groups to receive daily intraperitoneal injection of 1) Olaparib (10mg/kg, MedChemExpress), 2) TEPP-46 (25mg/kg,

Cayman Chemical), and 3) vehicle (10% DMSO, 40% polyethylene glycol 300, 50% saline). Echocardiography was performed every week, beginning 3-weeks after PAB surgery in rats. Eight-weeks post PAB, rats underwent right heart catheterization before sacrifice. Mice were evaluated with echocardiography every week from before the surgery and euthanized 7-weeks after PAB.

Sugen/Hypoxia Animal Model

Adult mice (11 weeks old) were injected subcutaneously with the VEGFR2 antagonist Su5416 (Sugen, APExBIO) at 20mg/kg once a week for 3 weeks. After the first injection, mice were exposed to 10% oxygen (hypoxia) in a ventilated chamber for 3 weeks. All animals had access to standard mouse chow and water. Animals were housed under a 12-hours light/dark cycle under controlled temperature conditions.

Echocardiographic and Hemodynamic Measurements

Transthoracic echocardiography was performed using a Vevo 3100 (Visualsonics) under inhalation of isoflurane. Left ventricle eccentricity index (EI) was measured at the level of papillary muscles in B-mode tracings. S', TAPSE, RV FAC, CO and CI were measured as previously described (4) and according to current guidelines (5). Closed-chest RV catheterization was performed under general anesthesia in all animals. A polyethylene catheter connected to a pressure transducer (SciSence) was inserted into the right external jugular vein, and threaded into the RV to obtain RV systolic and end-diastolic pressures and RV cardiac output, as previously described (4,6)

Right Ventricular Hypertrophy

After sacrifice, hearts were excised and the mass ratio between the RV and the left ventricle plus septum (Fulton index) was calculated as a measure of RV hypertrophy.

Cell Culture and Treatments

Rat H9c2 were purchased from ATCC and cultured as recommended. RV CMs were isolated by enzymatic dissociation from neonatal Sprague Dawley rats (2-3-days old), as previously described (4). CMs were also isolated from WT or *Parp1* knockout neonatal mice (2-3-days old) using the same protocol. Cells prepared in this way were resuspended in DMEM supplemented with 10%

fetal bovine serum (FBS, Gibco) and plated on tissue culture plastic for 90-minutes to remove non-CM cells. CMs were seeded on gelatin-coated dishes. On the following day, myocyte cultures were washed and refed with maintenance medium prior to incubation for up to 4-days.

Adult rat cardiomyocytes were isolated using an adapted Langendorff method described by O'Connel, Rodrigo and Simpson (7). Briefly, Sprague Dawley (250-350g) rats were injected intra-peritoneally with heparin (2U/g) 5min prior to anesthesia with isoflurane. The heart was carefully extracted from the rat with the aorta intact and placed in a conical tube containing Perfusion Buffer (120.4mM NaCl, 14.7mM KCl, 0.6mM KH_2PO_4, 0.6mM Na_2HPO_4, 1.2mM $MgSO_4$-$7H_2O$, 10mM Na-HEPES, 4.6mM $NaHCO_3$, 30mM Taurine, 10mM BDM and 5.5mM Glucose, pH 7.0). Extraneous tissue was removed and the aorta was cannulated with a 3mm cannula hanging from a Langendorff apparatus. Once cannulated, the heart was perfused with Perfusion Buffer (37°C) for 3min at 8mL/min to flush blood from the vasculature and remove extracellular calcium to stop contractions. The solution was then switched to myocyte digestion buffer (50mL of perfusion buffer containing 2.4mg/mL of collagenase type II and 30 mM $CaCl_2$). The heart was perfused with digestion Buffer for 20- to 25-minutes until the color became very pale and the tissue was soft. Once the digestion was complete, the heart was exposed to stopping buffer (perfusion buffer with 10% FBS and 12.5 mM $CaCl_2$). After a few minutes of manual gentle dissociation, any remaining large pieces of tissue were removed, and the tissue suspension was centrifuged for 3 minutes at 20g. Supernatant containing non-myocytes and dead myocytes were removed and the pellet containing viable cardiomyocytes was resuspended in Stopping Buffer supplemented with 2mM of ATP. The myocytes were passed through a series of increasing calcium concentrations by re-suspending centrifuged pellets in 100mM, 400mM, then 900mM $CaCl_2$ in Stopping Buffer. The final pellet was re-suspended in 10mL plating media (MEM media with 10% FBS, 10mM BDM, 2mM glutamine, 2mM ATP, and 1% Antibiotic-Antimycotic). Cardiomyocyte number and shape were assessed using a hemacytometer and Trypan Blue stain. Rod-shaped cardiomyocytes were plated on Laminin coated surfaces then incubated at 37 °C with 2% CO_2. After 1 hour, the plating

media was removed and replaced by culture media (MEM media with 0.1% BSA, 1% Anti-Anti, 10mM BDM, 2mM glutamine, 10mM ITS (Insulin-Transferrin-Selenium)). All experiments were completed within 36 hours after isolation.

After 24 hours of serum starvation, CMs were transiently transfected with rat PARP1 siRNA (50nM ID#197651, ThermoFisher Scientific) or corresponding control siRNA (siSCRM) using Lipofectamine RNAiMAX (Invitrogen) according to the manufacturer's protocol. To mimic the stressful environment CMs face during RV remodeling, cells were treated with endotheline-1 (ET-1, 100nmol/L, Abcam) for 24- to 48 hours before exposure to either hydrogen peroxide (H_2O_2, 300μM, Sigma-Aldrich) for 15 minutes or lipopolysaccharide (LPS, 1μg/ml, Sigma-Aldrich) for 12 hours. ABT-888 and Olaparib (two pharmacological inhibitors of PARP1) were purchased from Cedarlane and MedChemExpress, respectively. TEPP-46 and DASA-58 (two cytosolic activators/nuclear inhibitors of PKM2) were purchased from Cayman Chemical. PARP1 inhibitors, PKM2 activators or their vehicle (DMSO) were simultaneously applied with ET-1. At the end of treatments, cells were collected and analyzed.

Cardiomyocyte Subcellular Fractionation

For subcellular fractionation of H9c2, cells were suspended in a pH 7.9 lysis buffer containing 10mM HEPES, 1.5mM $MgCl_2$, 10mM KCl, 0.5mM DTT, 0.05% NP40 and protease inhibitor cocktail for 10-minutes at 4°C. The nuclei and other fragments were pelleted by centrifugation (3000rpm for 10-minutes) and supernatant was removed and kept as the cytosolic fraction. Nuclei were lysed by exposure to pH 7.9 high salt buffer containing 300mM NaCl, 5mM HEPES, 1.5mM MgCl2, 0.2mM EDTA, 0.5mM DTT and 26% glycerol for 30 minutes at 4°C and then exposed to sonication (20% amplitude, 20-seconds (1-second sonication+2-second intervals for 20 times)) followed by brief vortex mixes. Cellular debris were removed by centrifugation (13000rpm for 20-minutes at 4°C), and supernatants containing nuclear fractions were collected. Protein concentration was measured by Bradford assay. Separation between the nuclear and cytosolic

fractions was verified by blotting for the cytosolic protein α/β Tubulin and the nuclear protein Histone H3.

Mice/Rats Cardiac Fibroblasts Isolation and Treatment

Immediately after dissection, the free wall of the RV was dissected, placed in cold PBS and transferred to the cell culture room. The tissue was washed in HBSS twice and digested in a 2mL HBSS solution containing 350 U Collagenase II (Gibco) for 15-25 minutes at 37 °C with occasional manual agitation. Cold DMEM supplemented with 10% FBS was added to the cell digestion and resuspended several times using a 10mL serological pipet. The suspension was passed through 100μm mesh filters to remove debris and undigested tissue. After centrifugation at 250g for 5 minutes, the pellet was washed in PBS, centrifuged again, then resuspended in fibroblast growth medium with 2% FBS and provided supplements (Lonza). Isolated fibroblasts were stimulated with TGF-β1 (5ng/ml, Abcam) for 48 hours in presence or absence of PARP1/PKM2 modulators.

RNA Extraction and Quantitative Real-Time PCR

Quantitative RT-PCRs were performed as previously described (4). Briefly, total RNA from RV tissues was extracted using Trizol (Invitrogen), according to the manufacturer's instructions. Synthesis of cDNA was achieved using the qScript Flex cDNA Synthesis Kit (Quanta Bio) using 1 μg of RNA. Quantitative PCR was performed in triplicate on the QuantStudio 7 Flex real-time PCR system (Applied Biosystems). The exclusive amplification of the expected PCR product was confirmed by melting curve analysis and gel electrophoresis. The primers used are presented in Supplemental Table 4. The relative expression level of each target was determined by the ΔΔCt method. 18S ribosomal RNA was used as a reference.

Western Blot

Total proteins were extracted from human and rat/mouse right ventricle using a 2% Chaps protein extraction buffer supplemented with a protease-inhibitor cocktail (Roche). Protein concentration was determined using the Bradford method. Equal amounts of protein were loaded on SDS page,

electrophoresed, and transferred onto polyvinylidene fluoride membranes using a liquid blotting system (Bio-Rad). After blocking with either 5% non-fat dry milk or 5% goat serum in TBS-T buffer for 1 hour, membranes were incubated overnight at 4°C with indicated primary antibodies (Supplemental Table 5). Membranes were then incubated with appropriate HRP-conjugated secondary antibodies for 2-hours. Antibodies were revealed using ECL reagents (Perkin–Elmer) and using the imaging Chemidoc MP system (Bio-Rad). Protein expression was quantified using the Image lab software (Bio-Rad) and normalized to Amido black, as previously reported (8,9).

Histopathology and Immunohistochemistry

Human, rat and mouse RVs were fixed with 4% formaldehyde. Paraffin-embedded RVs were sectioned at 5μm, incubated in xylene to remove the paraffin and rehydrated through graded alcohols to water before staining with hematoxylin-eosin (for RV hypertrophy) and Masson-trichrome (for fibrosis). CM surface area (CSA) was obtained by tracing the outlines of CMs with a clear nucleus image in hematoxylin-eosin stained, as previously published (4). The quantification of RV fibrosis and CSA of CMs were determined in at least 10 randomly chosen areas with ImageJ software.

Lung tissue sections were stained with Elastica van Gieson (EVG) to identify and quantify distal PAs (< 75μm in diameter) wall thickness. PA wall thickness was calculated by averaging the external and internal diameter of transversally cut vessels and determining the mean distance between the lamina elastica externa and lumen in two perpendicular directions as previously described (8,10). For each specimen, at least 15 PAs were randomly outlined by an observer blinded to treatment.

For immunofluorescence staining, deparaffined and rehydrated tissue sections were subjected to an antigen retrieval procedure with heating 10mM citrate buffer (pH 6.0) in a pressure cooker for 15 minutes. Afterwards, sections were rinsed in PBS and blocked with 10% goat serum for 1h. After blocking tissue sections were incubated overnight at 4°C with the primary antibody (Supplemental Table 5). After three washing steps, appropriate fluorescent-conjugated secondary antibodies were

applied for 1 hour at room temperature. The slides were mounted in DAPI containing FluoroShield mounting media (Abcam). Images were acquired with a Zeiss Axio observer microscope using Zen software.

TUNEL and 8-oxodG staining

To assess myocytes apoptosis *in vitro* and *in vivo*, we used *in situ* cell death detection kit, TMR red (Sigma-Aldrich) according to the manufacturer's instructions. Cells or tissue sections were incubated with proteinase K (10mg/L) for 10-minutes following incubation with TdT-mediated dUTP-X nick end labeling (TUNEL) reaction buffer for 1 hour at 37°C. To investigate oxidative DNA damage *in vitro*, cytosoloc 8-Oxoguanine (8-oxodG) staining was performed as previously described (11). Briefly, cells were fixed in 2% paraformaldehyde (PFA) for 15 minutes at room temperature, then fixed in cold methanol for 10-minutes. Cells were then treated with RNAase at 37°C for 1 hour to eliminate RNA. After rinsing in PBS + 0.1% Triton-X 100, cells were re-fixed by incubation with 4% PFA for 10-minutes at room temperature and then mitochondrial DNA was denatured with 50mM NaOH in 50% ethanol for 5 minutes. Next, cells were incubated with blocking buffer (PBS with 10% goat serum) for 1 hour at room temperature before incubation overnight at 4°C with anti-8-hydroxy-2'deoxyguanosine mouse monoclonal antibody diluted in 2% goat serum. After washing with PBS, appropriate fluorescent secondary antibody was applied for 1 hour at RT. The coverslips were finally mounted on glass slides using DAPI fluoromount G mounting medium. Images were acquired with a Zeiss Axio observer microscope using Zen software.

Supplemental Tables

Supplemental Table 1. Clinical information of human RV samples.

Category	Origin	Diagnosis	Age	Sex	WHO class§	LVEF‖ (%)	RVSP‖ (mmHg)	TAPSE (mm)	CI‖ ($L/min/m^2$)	Interval between last CI‖ recorded and tissue sampling (days)	ERA	PDE5 inhibitor	Epoprostenol
CTRL	Biopsy	Aortic valve stenosis	56	M	N/A	60	N/A	25	2.77	56	no	no	no
CTRL	Biopsy	Aortic regurgitation	46	F	N/A	60	16	26	3.09	72	no	no	no
CTRL	Biopsy	Aortic valve stenosis	52	F	N/A	55	15	N/A	2.53	204	no	no	no
CTRL	Autopsy*	Sudden death	43	F	N/A	N/A	N/A	N/A	N/A	N/A	N/A	N/A	N/A
CTRL	Autopsy*	Sudden death	48	F	N/A	N/A	N/A	N/A	N/A	N/A	N/A	N/A	N/A
CTRL	Autopsy*	Sudden death	49	F	N/A	N/A	N/A	N/A	N/A	N/A	N/A	N/A	N/A
CTRL	Autopsy*	Sudden death	52	F	N/A	N/A	N/A	N/A	N/A	N/A	N/A	N/A	N/A
CTRL	Autopsy*	Sudden death	29	M	N/A	N/A	N/A	N/A	N/A	N/A	N/A	N/A	N/A
CTRL	Autopsy*	Sudden death	65	M	N/A	N/A	N/A	N/A	N/A	N/A	N/A	N/A	N/A
CTRL	Autopsy*	Sudden death	68	M	N/A	N/A	N/A	N/A	N/A	N/A	N/A	N/A	N/A
CTRL	Biopsy	Aortic valve stenosis	36	F	N/A	65	22	N/A	2.67	395	no	no	no
CTRL	Biopsy	Aortic valve stenosis	53	M	N/A	N/A	N/A	N/A	N/A	N/A	no	no	no
CTRL	Biopsy	Aortic valve stenosis	41	F	N/A	65	17	N/A	3.58	163	no	no	no
CTRL	Biopsy	Aortic valve stenosis	29	M	N/A	60	25	N/A	2.79	37	no	no	no
CTRL	Biopsy	Aortic valve stenosis	47	M	N/A	60	29	23	N/A	25	no	no	no
CTRL	Biopsy	Aortic valve stenosis	50	M	N/A	60	30	N/A	3.36	63	no	no	no
CTRL	Biopsy	Aortic valve stenosis	22	M	N/A	65	20	N/A	2.52	15	no	no	no
CTRL	Biopsy	Aortic valve stenosis	58	M	N/A	55	N/A	N/A	3.67	84	no	no	no
CTRL	Biopsy	Aortic valve stenosis	59	M	N/A	60	20	28	2.52	63	no	no	no
CTRL	Biopsy	Aortic valve stenosis	46	M	N/A	60	23	23	2.61	107	no	no	no
cRV	Biopsy	Pulmonary valve regurgitation†	29	M	N/A	60	35	18	2.54	8	no	no	no
cRV	Biopsy	Pulmonary valve stenosis‡	27	M	N/A	55	51	10	2.30	6	no	no	no
cRV	Autopsy	RV dilatation with unknow cause	30	F	II	60	42	16	3.00	4	no	no	no

Category	Origin	Diagnosis	Age	Sex	WHO class[§]	LVEF[‖] (%)	RVSP[‖] (mmHg)	TAPSE (mm)	CI[‖] (L/min/m^2)	Interval between last CI[‖] recorded and tissue sampling (days)	ERA	PDE5 inhibitor	Epoprostenol
cRV	Biopsy	Ventricular septal defect	61	F	N/A	60	65	17	3.00	53	no	no	no
cRV	Biopsy	Pulmonary valve regurgitation[†]	22	M	N/A	48	N/A	17	3.00	471	no	no	no
cRV	Biopsy	Pulmonary valve regurgitation[†]	33	F	N/A	50	21	18	4.10	378	no	no	no
cRV	Biopsy	Pulmonary valve stenosis[‡]	54	M	I-II	55	89	20	2.58	4	no	no	no
cRV	Biopsy	Pulmonary valve stenosis[‡]	14	F	N/A	66	N/A	N/A	2.25	N/A	no	no	no
cRV	Biopsy	Pulmonary valve stenosis[‡]	32	F	N/A	55	78	16	3.00	315	no	no	no
cRV	Biopsy	Ebstein anomaly	30	F	N/A	60	19	N/A	3.70	527	no	no	no
cRV	Biopsy	Pulmonary valve stenosis[‡]	27	M	N/A	60	97	N/A	2.50	196	no	no	no
cRV	Biopsy	Pulmonary valve regurgitation[†]	25	F	N/A	55	53	17	3.70	51	no	no	no
dRV	Autopsy	SSc-PAH	54	F	IV	60	60	17	1.51	233	yes	yes	no
dRV	Autopsy	SSc-PAH	77	M	III	50	44	13	1.7	363	yes	yes	no
dRV	Autopsy	SSc-PAH	47	F	III	60	80	12	0.7	17	yes	yes	yes
dRV	Autopsy	IPAH	74	F	IV	55	84	N/A	2.03	667	yes	no	no
dRV	Autopsy	PoPH	46	F	IV	80	115	N/A	N/A	2	no	no	no
dRV	Autopsy	SSc-PAH	53	F	IV	55	110	N/A	1.96	124	yes	yes	no
dRV	Autopsy	IPAH	76	F	IV	60	66	16	2.58	71	no	no	no
dRV	Autopsy	IPAH	61	F	III	45	100	16	1.73	7	yes	yes	no
dRV	Autopsy	IPAH	65	M	III	60	100	N/A	1.73	162	yes	yes	no
dRV	Autopsy	CHD-PAH	57	F	III	60	53	15	1.74	174	no	no	no
dRV	Autopsy	PVOD	72	F	IV	65	108	13	2.55	1142	no	no	no

SSc: systemic sclerosis; IPAH: Idiopathic pulmonary arterial hypertension; PoPH: portopulmonary hypertension; PVOD: pulmonary veno-occlusive disease; CHD: congenital heart disease; RV, right ventricle; cRV: compensated RV; dRV, decompensated RV; N/A: not available; LVEF: left ventricular ejection fraction; RVSP: RV systolic pressure; CI: cardiac index (L/min/m^2); ERA: endothelin receptor antagonist; PDE5: phosphodiesterase-5; TAPSE, tricuspid annular plane systolic excursion; WHO class, World Health Organization functional class.[2]

* Early autopsy performed following sudden death. None of those subjects had any past medical history. Moreover, there were no signs of acute or chronic right or left heart dysfunction. All subjects exhibited normal heart size, no heart hypertrophy (left and right ventricle wall diameter of 1.3-1.5 cm and 0.2-0.5 cm, respectively) and normal pulmonary arteries.

† Pulmonary valve regurgitation following previous surgical correction of tetralogy of Fallot.

‡ Pulmonary valve stenosis following previous surgical correction of tetralogy of Fallot. Elevated RV systolic pressure is related to transpulmonary valve gradient rather than pulmonary hypertension.

Biopsy performed during cardiac surgery.

§ WHO functional class assessed at the time of last clinic visit.

|| Measured by echocardiography.

Supplemental Table 2. Summary of human RV samples.

	CTRL (n=20)	cRV (n=12)	dRV (n=11)	P value
Age, (years)	47.5±11.8	32.0±13	62.0±11.5	0.0002
Female (%)	8 (40.0%)	8 (66.7%)	9 (81.8%)	0.06
BMI	28.2±6.6	27.3±4.7	24.5±4.6	0.46
Last CI (L/min/m^2) recorded[||]	2.89±0.43	2.97±0.59	1.83±0.56	0.01
Last LVEF (%) recorded[||]	60.4±3.3	57.0±4.9	59.1±8.9	0.23
Last TAPSE recorded (mm)[||]	25±2.1	16.6±2.7	14.6±1.9	<0.0001
Last RVSP recorded (mmHg)[||]	21.7±5.2	55.0±27	83.6±24.9	<0.0001
Interval (days) between last echo and biopsy or autopsy	107±106	183±204	269±348	0.71
**Diagnosis (%)**				
Sudden death[*]	7 (35%)	-	-	
Aortic valve stenosis	13 (65%)	-	-	
IPAH	-	-	4 (36.4%)	
CHD	-	11 (91.7%)	1 (9.1%)	
CTD-PAH	-	-	4 (36.4%)	
Pulmonary veno-occlusive disease	-	-	1 (9.1%)	
Portopulmonary Hypertension	-	-	1 (9.1%)	
Unknown cause	-	1 (8.3%)	-	
**WHO class (%)**				
II	-	2 (16.7%)	-	
III	-	-	5 (45.5%)	
IV	-	-	6 (54.5%)	
**PAH therapy (%)**				
ERA	-	-	7 (63.6%)	
PDE5 inhibitor	-	-	6 (54.5%)	
Epoprostenol	-	-	1 (9.1%)	

Values are presented with means±SD. BMI: body mass index; CI: cardiac index (L/min/m^2); LVEF: left ventricular ejection fraction; TAPSE, tricuspid annular plane systolic excursion; RVSP: RV systolic pressure; IPAH: Idiopathic pulmonary arterial hypertension; CHD: congenital heart disease; CTD: connective tissue disease; WHO class, World Health Organization functional class; ERA: endothelin receptor antagonist; PDE5: phosphodiesterase-5. Comparisons were made

using One way ANOVA followed by Tukey's multiple comparison tests, non-parametric Kruskal-Wallis tests or chi-square tests. [||] Measured by echocardiography.

[*] Early autopsy was performed following sudden death. None of those subjects had any past medical history. Moreover, there were no macroscopic signs of chronic right nor left heart dysfunction. All subjects exhibited normal heart size, no heart hypertrophy (left and right ventricle wall diameter of 1.3-1.5 cm and 0.2-0.5 cm, respectively) and normal pulmonary arteries.

Supplemental Table 3. Categorization of rats in the monocrotaline (MCT) and pulmonary artery banding (PAB) models of RV failure.

Model	Group	Interval between MCT injection or sham/PAB surgery and sacrifice (days)	Criteria	Hemodynamics before collecting samples		
				RVSP† (mmHg)	CO (ml/min)	RVEDP† (mmHg)
MCT rat‡	CTRL	NA	rats that received saline	25.3 ± 5.9	104.5 ± 17.0	1.8 ± 0.8
	cRV	16 ± 8	PH without an end-stage of RV failure sign* and with preserved CO (CO > 61.3 ml ml/min) † without elevated RVEDP (RVEDP < 7.7 mmHg)	63.3 ± 10.0	84.6 ± 13.6	3.8 ± 1.6
	dRV	27 ± 6	PH without an end-stage of RV failure sign* and met one of the following criteria: 1) reduced CO (CO < 61.3 ml/min) † without elevated RVEDP (RVEDP < 7.7 mmHg) or 2) elevated RVEDP (RVEDP > 7.7 mmHg)	73.2 ± 6.9	56.6 ± 9.3	7.4 ± 2.9
PAB rat§	Sham	34 ± 19	rats that underwent thoracotomy alone	-	150.1 ± 18.1	-
	cRV	23 ± 12	PAB rats without any end-stage of RV failure sign* and with preserved CO (CO ≥ 90.4 ml ml/min) #	-	126.6 ± 26.6	-
	dRV	36 ± 7	PAB rats with end-stage of RV failure signs* or reduced CO (CO < 90.4 ml/min) #	-	69.7 ± 10.4	-

In MCT rat RVs, the cut-off values of cRV and dRV were determined as previously described (4). In PAB rat RVs, cut-off values of cRV and dRV were set as median CO measured by echocardiography. Data are expressed as mean ± SD.

* end-stage of RV failure sign: death or severe clinical symptoms (ruffled fur, red discoloration of head and neck, less active than previously and decreased food consumption defined as less than 5 g per day).

† Measured by closed-chest right heart catheterization.

# Measured by echocardiography.

Supplemental Table 4. Primer sequences or references used for SYBR green or Taqman assays.

TaqMan Assay (ThermoFisher Scientific)	
Rat *Nppa*	Rn00664637_g1
Rat *Nppb*	Rn00580641_m1
Mouse *18s* rRNA	4310893E
SYBR Green Primers	
Human *Col1α1* (forward 5'-3')	TTCTGTACGCAGGTGATTGG
Human *Col1α1* (reverse 5'-3')	GACATGTTCAGCTTTGTGGAC
Human *Col3α1* (forward 5'-3')	TTGGCATGGTTCTGGCTT
Human *Col3α1* (reverse 5'-3')	CTACTTCTCGCTCTGCTTCATC
Human *Ccl2* (forward 5'-3')	GCCTCTGCACTGAGATCTTC
Human *Ccl2* (reverse 5'-3')	AGCAGCCACCTTCATTCC
Human *Il-8* (forward 5'-3')	CATCTTCACTGATTCTTGGATACC
Human *Il-8* (reverse 5'-3')	TGTCTGGACCCCAAGGAA
Human *Socs3* (forward 5'-3')	CAAGGACGGAGACTTCGATTC
Human *Socs3* (reverse 5'-3')	GGAAACTTGCTGTGGGTGA
Human *18s* rRNA (forward 5'-3')	CGCACGGCCGGTACAGTGAA
Human *18s* rRNA (reverse 5'-3')	GGGAGAGGAGCGAGCGACCA
Rat *Col1α1* (reverse 5'-3')	CGCAAAGAGTCTACATGTCTAGG
Rat *Col1α1* (forward 5'-3')	CATTGTGTATGCAGCTGACTTC
Rat *Col3α1* (forward 5'-3')	TCTCTAGACTCATAGGACTGACC
Rat *Col3α1* (reverse 5'-3')	TTCTTCTCACCCTGCTTCAC
Rat *Ccl2* (forward 5'-3')	GAATGAGTAGCAGCAGGTGAG
Rat *Ccl2* (reverse 5'-3')	ATCTCTCTTCCTCCACCACTA
Rat *Il-8* (forward 5'-3')	TGACAGCATAGCCTCAATGG
Rat *Il-8* (reverse 5'-3')	CTTTACAAGTCCGGTTTACTCCT
Rat *Myh6* (forward 5'-3')	TCATCCACGGCCAATTCTTG
Rat *Myh6* (reverse 5'-3')	CACCAGAATCCAGGCTCAA
Rat *Myh7* (forward 5'-3')	TCATGGACCTGGAGAACGAC
Rat *Myh7* (reverse 5'-3')	CCTGGCGTTGAGTGCATTT
Rat *Socs3* (forward 5'-3')	CCCGCTTTGACTCTGTACT
Rat *Socs3* (reverse 5'-3')	TCAGTACCAGCGGGATCTTCTC
Mouse *Col1α1* (forward 5'-3')	CGCAAAGAGTCTACATGTCTAGG
Mouse *Col1α1* (reverse 5'-3')	CATTGTGTATGCAGCTGACTTC
Mouse *Col3α1* (forward 5'-3')	TTCTTCTCACCCTTCTTCATCC
Mouse *Col3α1* (reverse 5'-3')	TCTCTAGACTCATAGGACTGACC
Mouse *Ccl2* (forward 5'-3')	CATCCACGTGTTGGCTCA
Mouse *Ccl2* (reverse 5'-3')	AACTACAGCTTCTTTGGGACA
Mouse *Il-1β* (forward 5'-3')	GACCTGTTCTTTGAAGTTGACG
Mouse *Il-1β* (reverse 5'-3')	CTCTTGTTGATGTGCTGCTG
Mouse *Il-6* (forward 5'-3')	AGCCAGAGTCCTTCAGAGA

Mouse *Il-6* (reverse 5'-3')	TCCTTAGCCACTCCTTCTGT
Mouse *Myh6* (forward 5'-3')	GCGCATTGAGTTCAAGAAGATAG
Mouse *Myh6* (reverse 5'-3')	AAGTAGAGCTTCATCCATGGC
Mouse *Myh7* (forward 5'-3')	CAACATGGAGCAGATCATCAAG
Mouse *Myh7* (reverse 5'-3')	CTGGTGAGGTCATTGACAGAA
Mouse *Nppa* (forward 5'-3')	TCTGATGGATTTCAAGAACCTGC
Mouse *Nppa* (reverse 5'-3')	ATCTATCGGAGGGGTCCCAG
Mouse *Nppb* (forward 5'-3')	GAGTCCTTCGGTCTCAAGGC
Mouse *Nppb* (reverse 5'-3')	CAACTTCAGTGCGTTACAGCC
Mouse *Socs3* (forward 5'-3')	GAGATTTCGCTTCGGGACTA
Mouse *Socs3* (reverse 5'-3')	GGAAACTTGCTGTGGGTGA
Rat *Il-1β*	qRnoCID0004680 (Biorad)
Rat *Il-6*	qRnoCID0053166 (Biorad)
Rat *18s* rRNA	PPR57734E (Qiagen)

Supplemental Table 5. Primary antibodies used for Western blotting and histological analysis.

Primary antibody	Vendor	Catalog #	Blocking	Dilution	Molecular weight (kDa)
**Western blotting**					
CCL2/MCP-1	Abcam	25124	5% non fat dry milk	1 :1000	15
cMYC	Cell Signaling	5605	5% goat serum	1 :1000	60
CTGF	Abcam	6992	5% non fat dry milk	1 :1000	35-40
Fibronectin	Abcam	6328	5% non fat dry milk	1 :1000	270
H3	Cell Signaling	4499T	5% non fat dry milk	1 :2000	15-20
HK2	Cell Signaling	2867	5% non fat dry milk	1 :1000	100
IL-1β	Novus Biological	19775	5% non fat dry milk	1 :1000	25
IL-6	Abcam	9324	5% non fat dry milk	1 :1000	25
LDH(A/C)	Cell Signaling	3558S	5% non fat dry milk	1 :2000	35-40
MMP2	Cell Signaling	4022	5% non fat dry milk	1 :1000	65
NFκB	BD Bioscience	610868	5% non fat dry milk	1 :1000	65
NUDT1	Cell Signaling	43918	5% non fat dry milk	1 :1000	20
PARP1	Santa Cruz	7150	5% non fat dry milk	1 :1000	110-120
PKM1	Proteintech	15821	5% non fat dry milk	1 :2000	60
PKM2	Cell Signaling	3198	5% non fat dry milk	1 :1000	60
p(Tyr105)-PKM2	Cell Signaling	3827S	5% goat serum	1 :1000	60
αSMA	Abcam	5694	5% non fat dry milk	1 :1000	40-45
p(Tyr705)-STAT3	Cell Signaling	9131	5% non fat dry milk	1 :1000	80
STAT3	Cell Signaling	9139	5% non fat dry milk	1 :1000	80
α/β Tubulin	Cell Signaling	2148	5% non fat dry milk	1 :1000	50
**Histology**					
CD4	Santa Cruz Biotechnology	20079	10% Goat Serum	1 :200	-
CD8	Abcam	52853	10% Goat Serum	1 :200	-
CD68	Abcam	955	10% Goat Serum	1 :200	-
NFκB	BD Bioscience	610868	10% Goat Serum	1 :200	-
8-oxodG	Abcam	48508	10% Goat Serum	1 :50	-
PAR	Cell Signaling	83732	10% Goat Serum	1 :200	-
PARP1	Santa Cruz Biotechnology	7150	10% Goat Serum	1 :400	-
PARP1	Santa Cruz Biotechnology	56197	10% Goat Serum	1 :400	-
PKM2	Cell Signaling	3198	10% Goat Serum	1 :200	-
TroponinT	Abcam	8295	10% Goat Serum	1 :200	-

Supplemental Figure Legends

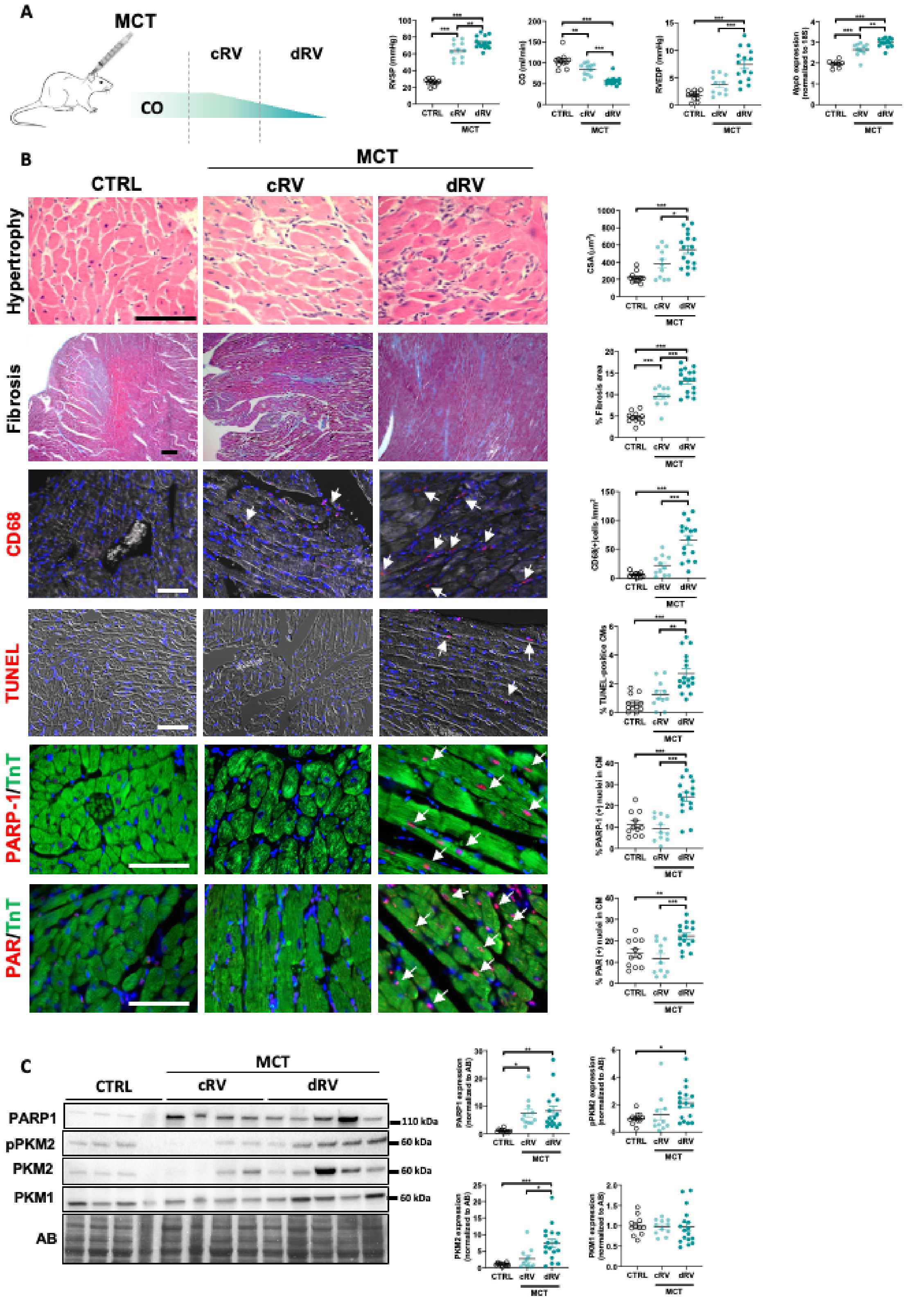

Supplemental Figure 1. Increased expression and activity of PARP1 and PKM2 in decompensated RV from MCT rats. (A) Schematic representation of categorization of MCT rats based on hemodynamics. MCT rats underwent right heart catheterization at different time points over a period of four weeks, starting 1 week after MCT injection. A group of control rats was used to defined normal RVSP, CO and RVEDP. MCT-injected rats were subsequently classified into cRV or dRV groups based on hemodynamic data and relative expression of *Nppb*. **(B)** Representative images and corresponding quantification of RV sections from control (n = 11) and MCT rats classified as cRV (n = 11) or dRV (n = 16) stained with H&E (CM surface area), Masson's trichrome (fibrosis), CD68 (infiltration of macrophages), TUNEL (apoptosis) or double-labeled for Troponin T and either PARP1 or PAR. **(C)** Representative Western blots and quantification of PARP1, pPKM2, PKM2, and PKM1 in RV from control and MCT rats. Scale bars: 50μm. Arrows: positive cells. *P<0.05, **P<0.01, ***P<0.001. Scatter dot plots show individual values and mean ± SEM. Comparisons were made using One-way ANOVA followed by Tukey's multiple comparison tests or the non-parametric Kruskal-Wallis tests.

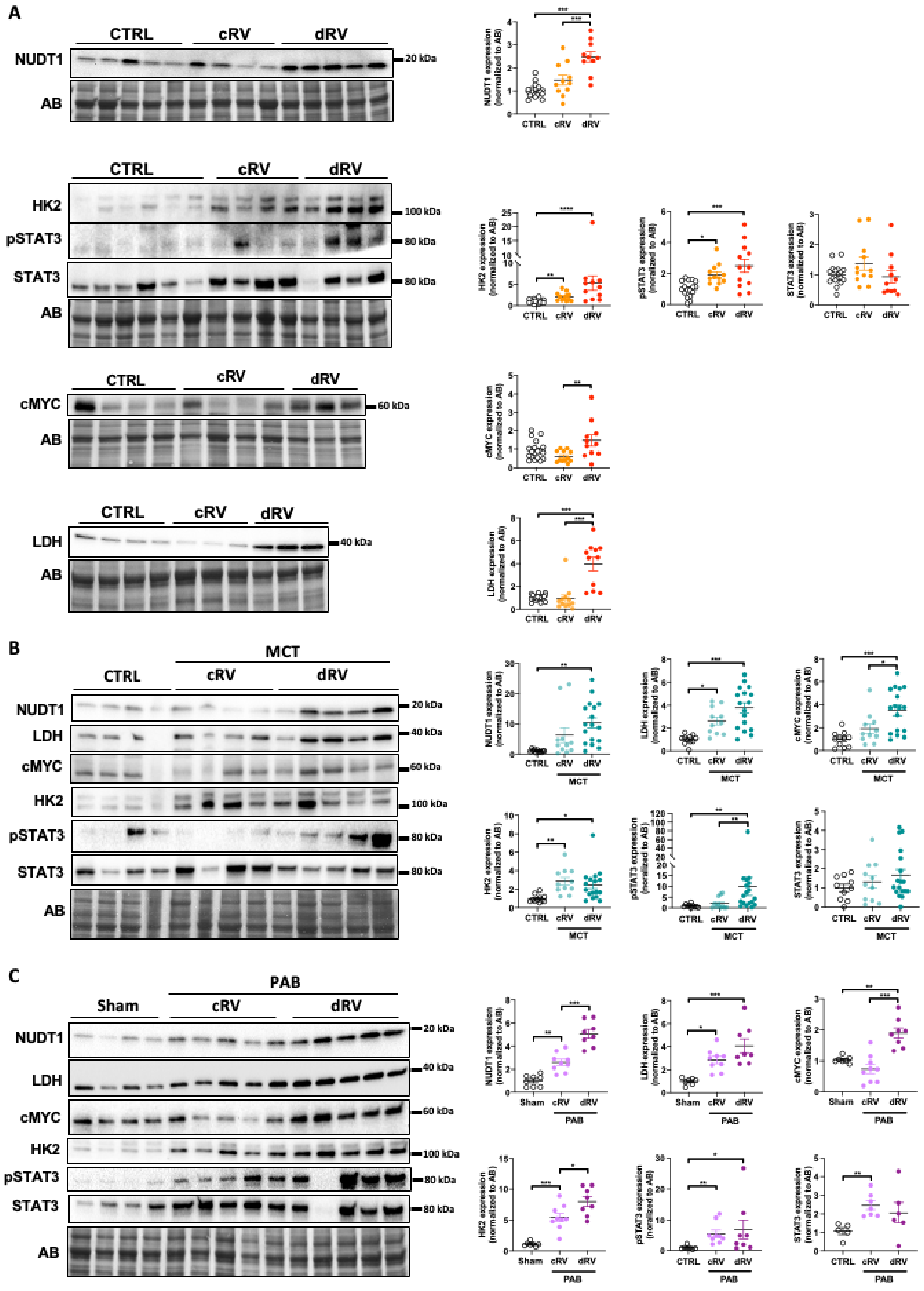

Supplemental Figure 2. Increased expression of glycolysis and oxidative DNA damage related proteins in decompensated RV from Human, MCT rats, and PAB rats. (A) Representative

Western blots and quantification of NUDT1, HK2, pSTAT3, STAT3, cMYC and LDH in human RV from controls, cRV and dRV PAH patients. Controls (n = 17), cRV (n = 12) and dRV (n = 11). **(B)** Representative Western blots and quantification of NUDT1, LDH, cMYC, HK2, pSTAT3 and STAT3 in RV from control and MCT rats. Control (n = 11), cRV (n = 11) and dRV (n = 17). **(C)** Representative Western blots and quantification of NUDT1, LDH, cMYC, HK2, pSTAT3 and STAT3 in RV from rats subjected to PAB or sham surgery. Sham (n = 7), cRV (n = 9) and dRV (n = 8). *P<0.05, **P<0.01, ***P<0.001. Scatter dot plots show individual values and mean ± SEM. Comparisons were made using One-way ANOVA followed by Tukey's multiple comparison tests or the non-parametric Kruskal-Wallis tests.

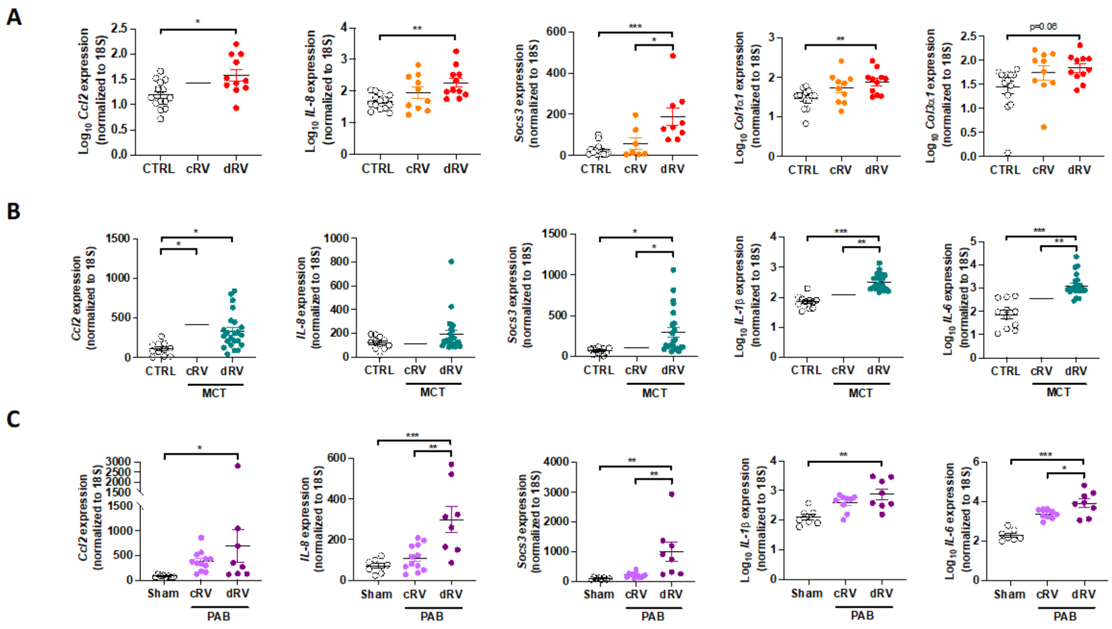

Supplemental Figure 3. Increased expression of inflammation related genes in decompensated RV from Human, MCT rats, and PAB rats. (A) Representative mRNA levels of *Ccl2*, *IL-8*, *Socs3*, *Col1α1* and *Col3α1* in human RV from controls, cRV and dRV PAH patients. Controls (n = 14), cRV (n = 10) and dRV (n = 11). **(B)** Representative mRNA levels of *Ccl2*, *IL-8*, *Socs3*, *IL-1β* and *IL-6* in RV from control and MCT rats. Control (n = 11), cRV (n = 10) and dRV (n = 23). **(C)** Representative mRNA levels of *Ccl2*, *IL-8*, *Socs3*, *IL-1β* and *IL-6* in RV from rats subjected to PAB or sham surgery. Sham (n = 7-8), cRV (n = 9-12) and dRV (n = 8). *P<0.05, **P<0.01, ***P<0.001. Scatter dot plots show individual values and mean ± SEM. Comparisons were made using One-way ANOVA followed by Tukey's multiple comparison tests or the non-parametric Kruskal-Wallis tests.

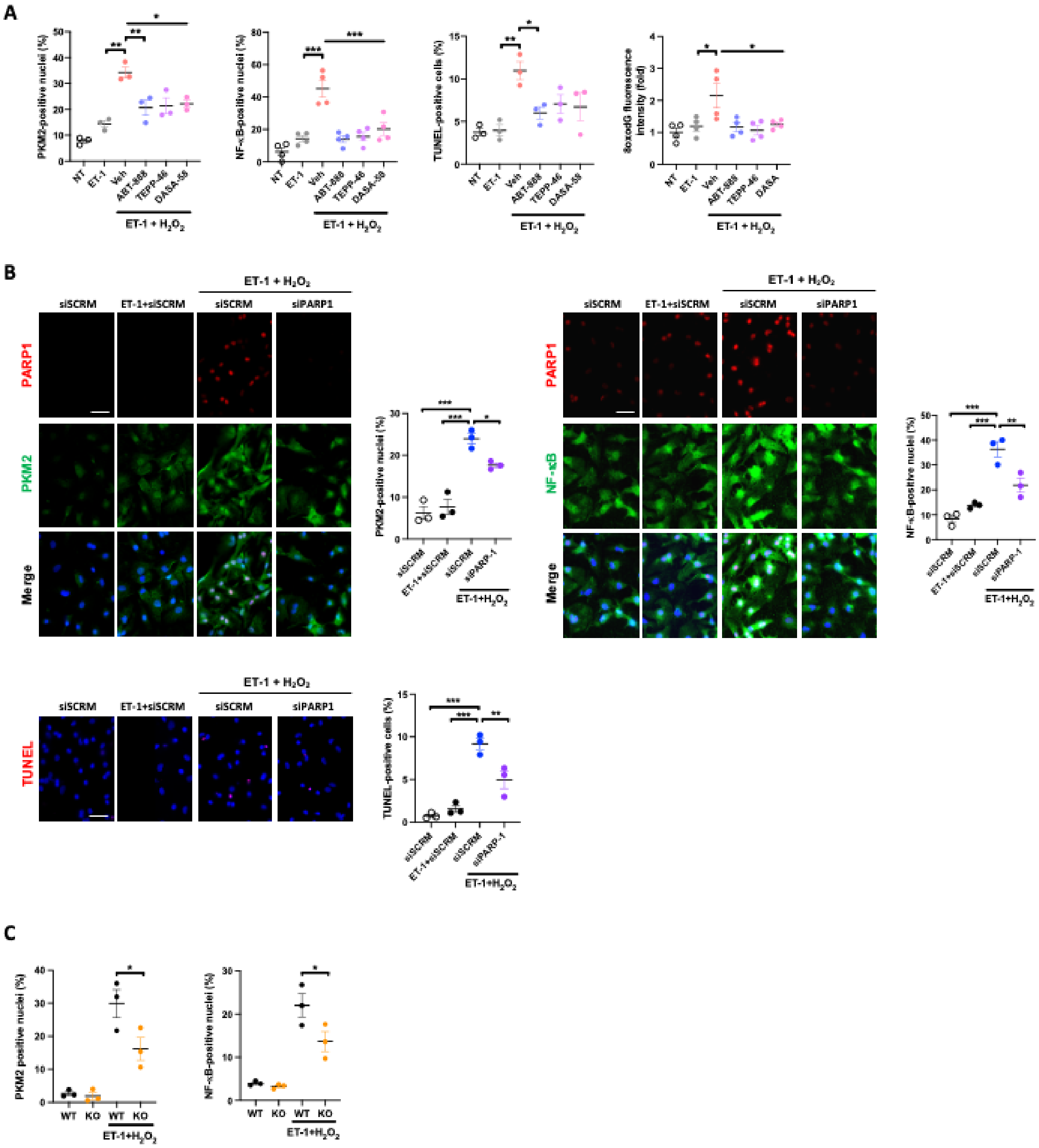

Supplemental Figure 4 (related to Figure 3). Inhibition of PARP1 as well as cytosolic retention of PKM2 prevent cardiomyocyte dysfunction. (A) Quantification of the percentage of neonatal rat RV CMs (n = 3) exhibiting nuclear staining of PKM2, NF-κB, and TUNEL and quantification of 8oxodG fluorescence intensity following exposure or not to either ET-1 or ET-1+H_2O_2 in presence or absence of ABT-888, TEPP-46 and DASA-58. **(B)** Representative fluorescent images and corresponding quantification of PARP1, PKM2, NF-κB, and TUNEL staining in neonatal rat

RV CMs (n = 3) transfected with siSCRM or siPARP1 and exposed or not to ET-1 or ET-1+H_2O_2. **(C)** Quantification of the percentage of *Parp1*$^{+/+}$ or *Parp1*$^{-/-}$ mouse CMs (n = 3) exhibiting nuclear staining of PKM2 and NF-κB following exposure or not to ET-1+H_2O_2. Scale bars: 25μm. *P<0.05, **P<0.01, ***P<0.001. Scatter dot plots show individual values and mean ± SEM. Comparisons were made using One-way ANOVA followed by Tukey's multiple comparison tests.

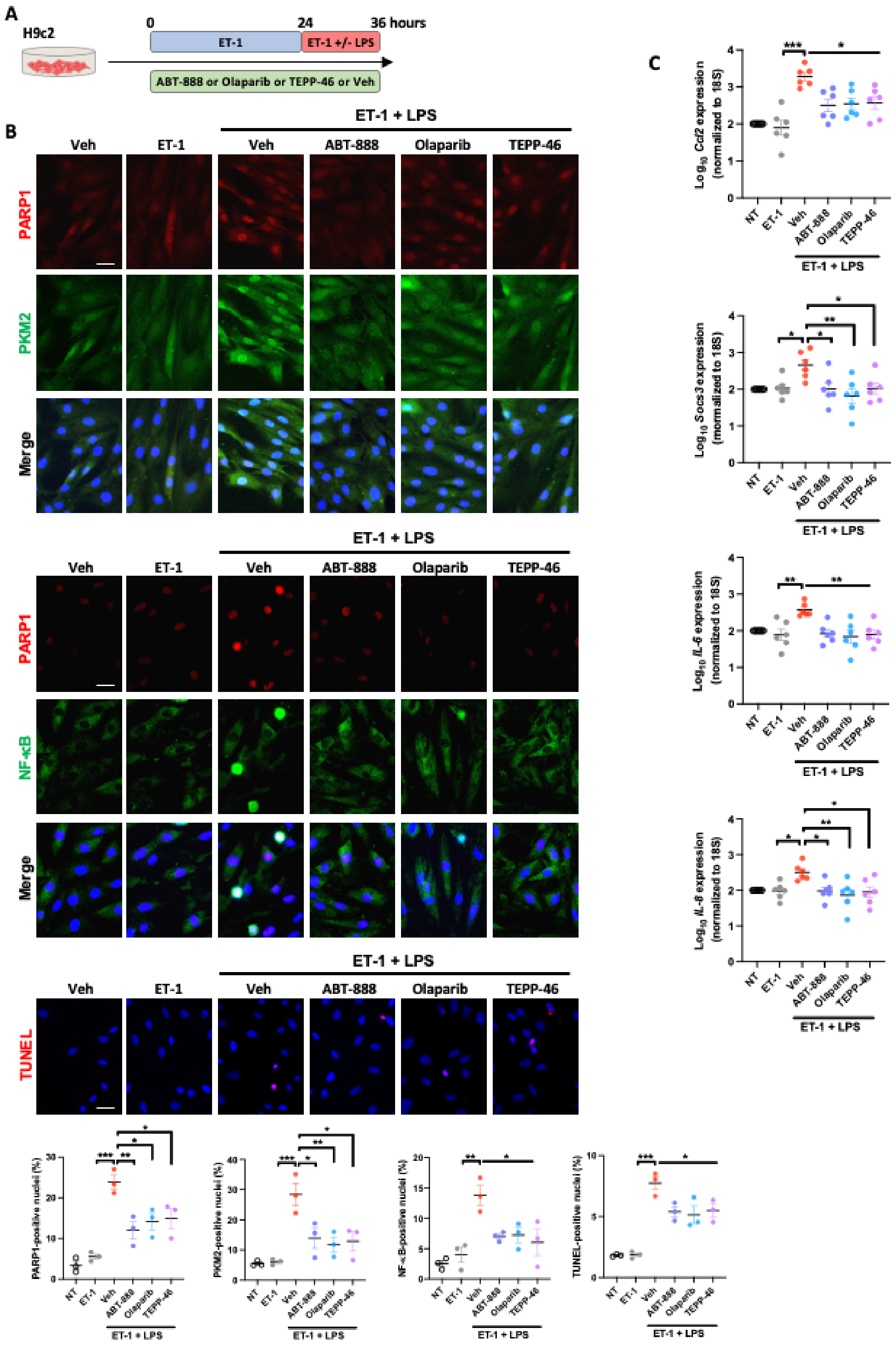

Supplemental Figure 5. Inhibition of PARP1 and cytosolic retention of PKM2 prevent H9c2 cells dysfunction under exposure to LPS. (A) Schematic of the experimental design. **(B)**

Representative immunofluorescent images of PARP1, PKM2, NF-κB and TUNEL in H9c2 exposed or not to either ET-1 or ET-1+LPS in presence or absence of ABT-888, Olaparib and TEPP-46. The quantification of the percentage of cells exhibiting nuclear staining of PARP1, PKM2, NF-κB, and TUNEL are presented. (n = 3) **(C)** Relative mRNA levels of *Ccl2*, *Socs3*, *IL-6* and *IL-8* in H9c2 exposed or not to either ET-1 or ET-1+LPS in presence or absence of ABT-888, Olaparib and TEPP-46. (n = 3) Scale bars: 25μm. *P<0.05, **P<0.01, ***P<0.001. Scatter dot plots show individual values and mean ± SEM. Comparisons were made using One-way ANOVA followed by Tukey's multiple comparison tests.

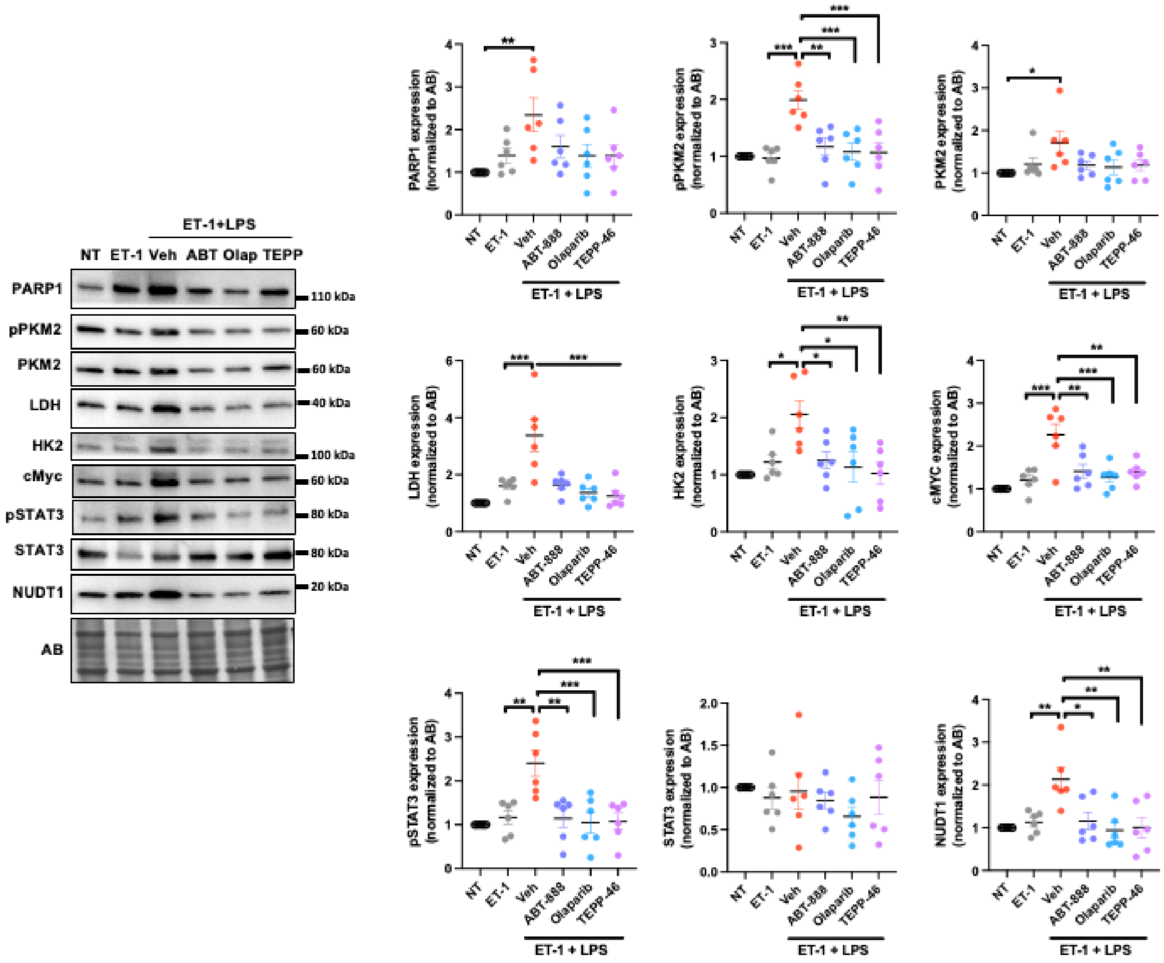

Supplemental Figure 6. Inhibition of PARP1 and cytosolic retention of PKM2 prevent increase of glycolysis and DNA damage related proteins under exposure to LPS and ET-1. Representative Western blots and quantification of PARP1, pPKM2, PKM2, LDH, HK2, cMYC, pSTAT3, STAT3 and NUDT1 in H9c2 exposed or not to either ET-1 or ET-1+LPS in presence or absence of ABT-888, Olaparib and TEPP-46. Western blots were executed with whole cell lysates (n = 6). *P<0.05, **P<0.01, ***P<0.001. Scatter dot plots show individual values and mean ± SEM. Comparisons were made using One-way ANOVA followed by Tukey's multiple comparison tests.

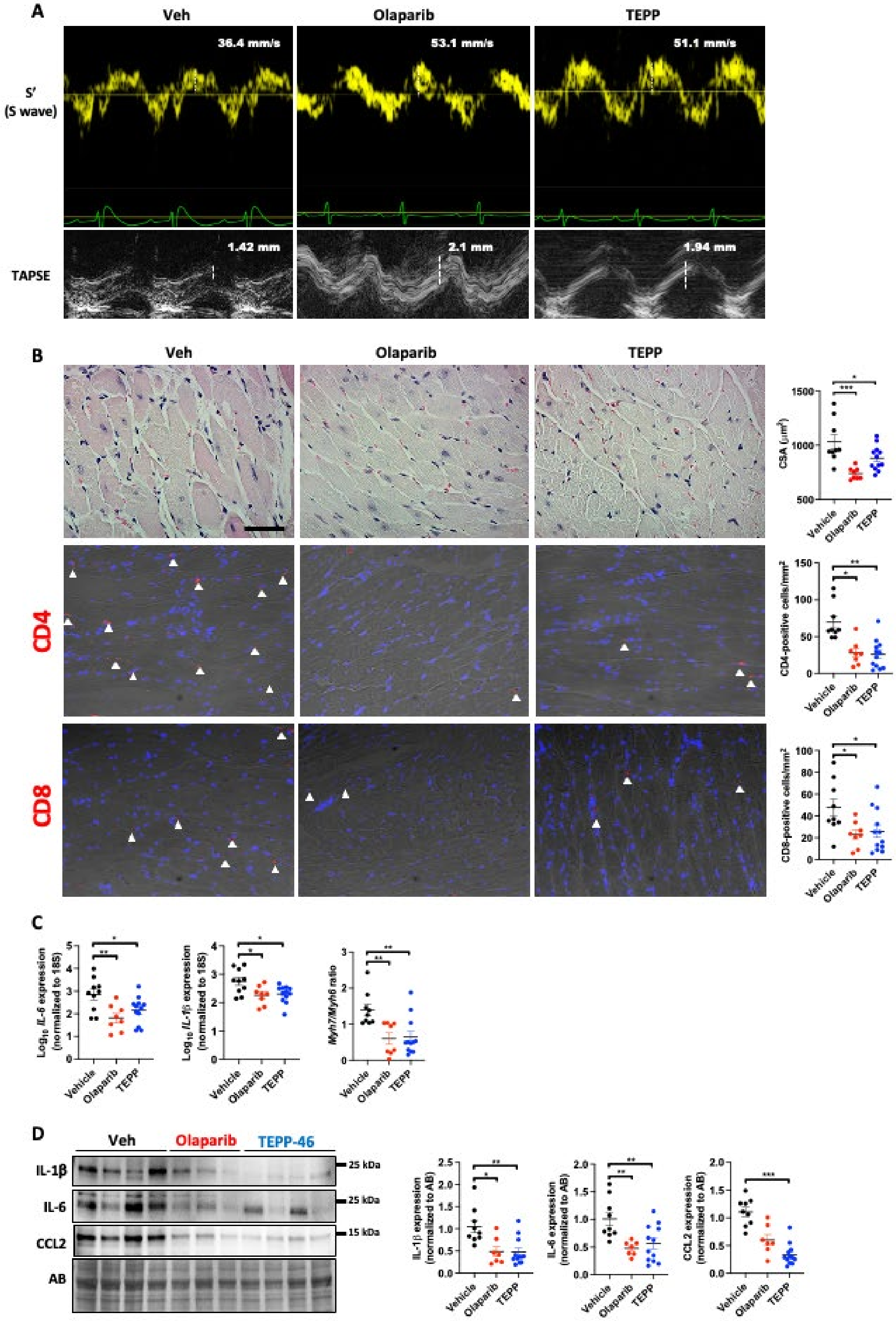

Supplemental Figure 7 (related to Figure 6). Cardioprotective effects of PARP1 inhibitor and PKM2 activator in rats subjected to PAB. (A) Representative echocardiographic images of S

wave and TAPSE in PAB rats treated with Olaparib, TEPP-46 or vehicle at six weeks after initiation of the treatment. **(B)** Representative images of H&E-stained RV sections and quantification of CM surface area in PAB rats treated with Olaparib, TEPP-46 or vehicle at six weeks after initiation of the treatment. Representative images and corresponding quantification of RV sections stained with CD4 and CD8 in PAB rats treated with Olaparib, TEPP-46 or vehicle at six weeks after initiation of the treatment. **(C)** Relative mRNA levels of *IL-6*, *IL-1β* and *Myh7/Myh6* ratio in PAB rats treated or not with Olaparib or TEPP-46. **(D)** Representative Western blots and quantification of IL-1β, IL6, and CCL2 in RV from PAB rats exposed or not to Olaparib or TEPP-46. Scale bars: 25μm. n = 8-12 per group. *P<0.05, **P<0.01, ***P<0.001. Scatter dot plots show individual values and mean ± SEM. Comparisons were made using One-way ANOVA followed by Tukey's multiple comparison tests.

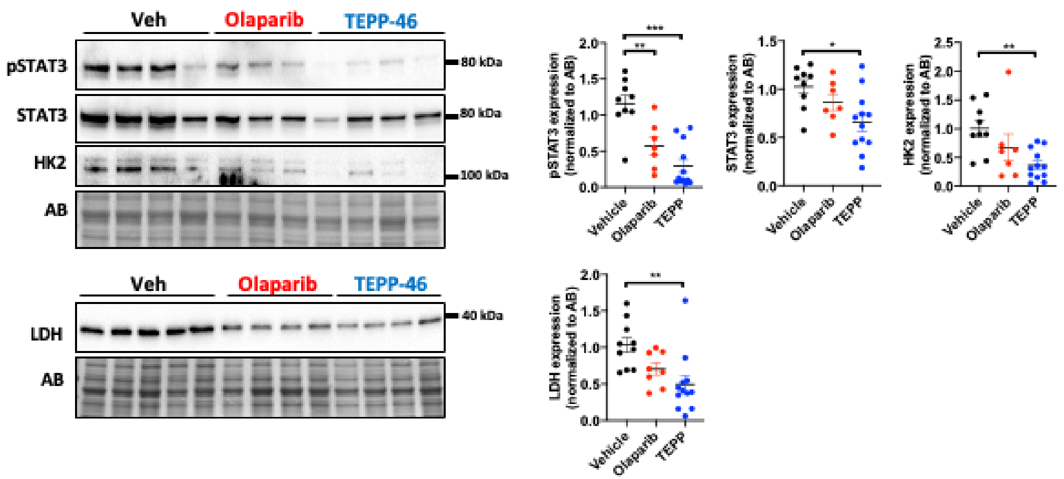

Supplemental Figure 8 (related to Figure 6). Cardioprotective effects of PARP1 inhibitor and PKM2 activator in rats subjected to PAB. Representative Western blots and quantification of pSTAT3, STAT3, HK2, and LDH in RV from PAB rats exposed or not to Olaparib or TEPP-46. n = 8-12 per group. *P<0.05, **P<0.01, ***P<0.001. Scatter dot plots show individual values and mean ± SEM. Comparisons were made using One-way ANOVA followed by Tukey's multiple comparison tests.

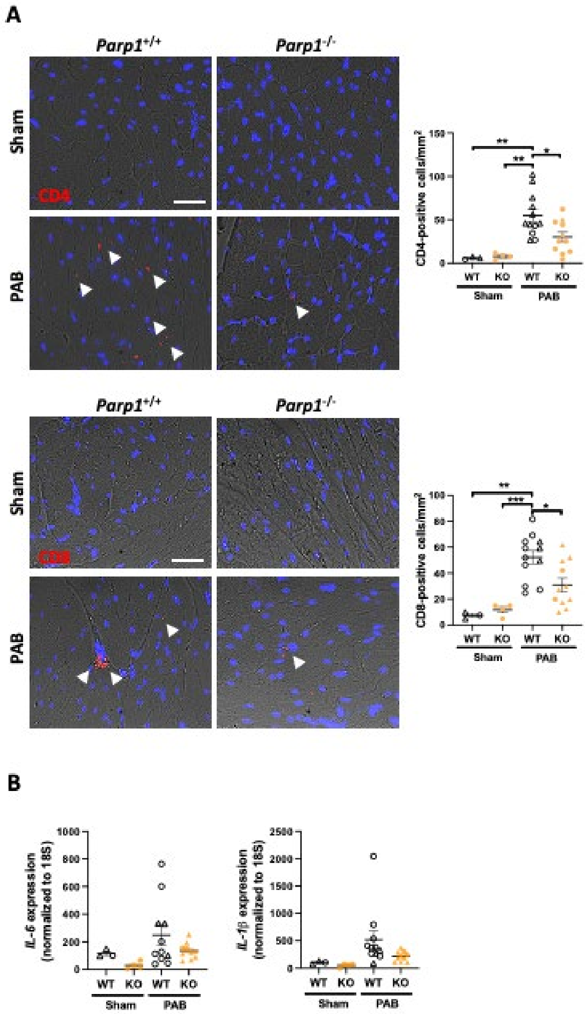

Supplemental Figure 9 (related to Figure 7). *Parp1* inactivation attenuates PAB-induced RV inflammation. (A) Representative images and corresponding quantification of RV sections stained with CD4 and CD8 in sham or PAB-operated wild-type and *Parp1* knockout mice. **(B**) Relative mRNA levels of *IL-6 and IL-1β* in RV of 7-week sham- of PAB-operated wild-type and *Parp1* knockout mice. Scale bars: 25µm. Data are presented as mean ± SEM. n = 3-11 per group. Female mice are indicated by triangular symbols, and male mice are indicated by circular symbols. *P<0.05, **P<0.01, ***P<0.001. Comparisons were made using One-way ANOVA followed by Tukey's multiple comparison tests.

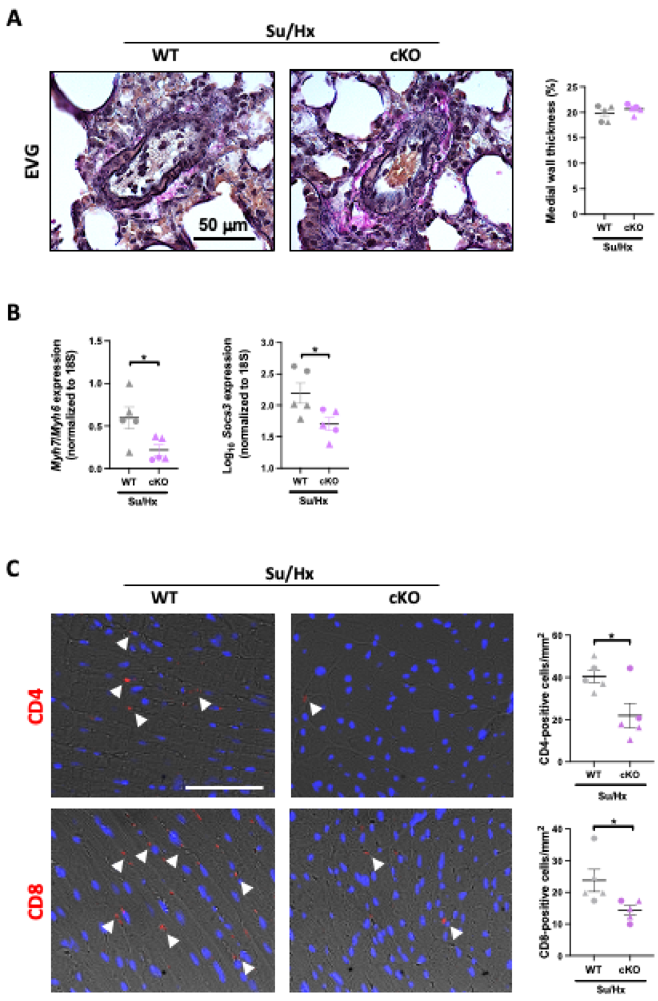

Supplemental Figure 10 (related to Figure 8). Cardiac specific *Parp1* deletion prevents Su/Hx-induced RV dysfunction regardless of pulmonary vascular remodeling. (A) Representative image of EVG-stained lung sections from *Parp1*$^{flox/flox}$ (WT) and *Parp1*$^{flox/flox}$;Tg$^{+/Myh6\text{-}Cre}$ (cKO) mice after exposure to Su/Hx and quantification of the percentage of PA (<75µm) medial wall thickness. **(B)** Relative mRNA levels of *Myh7/Myh6* ratio and *Socs3* in RV from Su/Hx-exposed WT and cKO mice. **(C)** Representative images and corresponding quantification of RV sections stained with CD4 and CD8 in Su/Hx-exposed WT and cKO mice. Scale bars: 50µm. Data are presented as mean ± SEM. n = 5 per group. Female mice are indicated by triangular symbols, and male mice are indicated by circular symbols. *P<0.05. Comparisons were made using unpaired Student t tests.

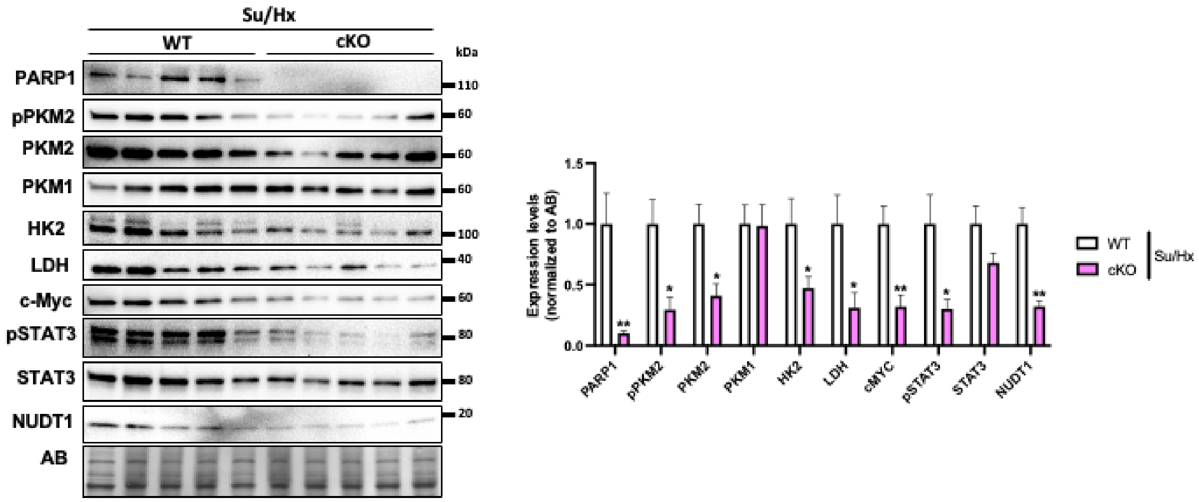

Supplemental Figure 11 (related to Figure 8). Cardiac specific *Parp1* deletion prevents Su/Hx-induced increase of glycolysis and DNA damage related proteins in RVs. Representative Western blots and quantification of PARP1, pPKM2, PKM2, PKM1, HK2, LDH, cMYC, pSTAT3, STAT3, and NUDT1 in RV from WT and *Parp1* cKO mice exposed to Su/Hx. Data are presented as mean ± SEM. n = 5 per group. *P<0.05, **P<0.01. Comparisons were made using unpaired Student t test.

Supplemental References

1. Wang ZQ, Auer B, Stingl L et al. Mice lacking ADPRT and poly(ADP-ribosyl)ation develop normally but are susceptible to skin disease. Genes & development 1995;9:509-20.
2. Luo X, Ryu KW, Kim DS et al. PARP-1 Controls the Adipogenic Transcriptional Program by PARylating C/EBPbeta and Modulating Its Transcriptional Activity. Molecular cell 2017;65:260-271.
3. Agah R, Frenkel PA, French BA, Michael LH, Overbeek PA, Schneider MD. Gene recombination in postmitotic cells. Targeted expression of Cre recombinase provokes cardiac-restricted, site-specific rearrangement in adult ventricular muscle in vivo. J Clin Invest 1997;100:169-79.
4. Omura J, Habbout K, Shimauchi T et al. Identification of Long Noncoding RNA H19 as a New Biomarker and Therapeutic Target in Right Ventricular Failure in Pulmonary Arterial Hypertension. Circulation 2020;142:1464-1484.
5. Augustine DX, Coates-Bradshaw LD, Willis J et al. Echocardiographic assessment of pulmonary hypertension: a guideline protocol from the British Society of Echocardiography. Echo Res Pract 2018;5:G11-G24.
6. Potus F, Ruffenach G, Dahou A et al. Downregulation of MicroRNA-126 Contributes to the Failing Right Ventricle in Pulmonary Arterial Hypertension. Circulation 2015;132:932-43.
7. O'Connell TD, Rodrigo MC, Simpson PC. Isolation and culture of adult mouse cardiac myocytes. Methods Mol Biol 2007;357:271-96.
8. Boucherat O, Chabot S, Paulin R et al. HDAC6: A Novel Histone Deacetylase Implicated in Pulmonary Arterial Hypertension. Sci Rep 2017;7:4546.
9. Boucherat O, Peterlini T, Bourgeois A et al. Mitochondrial HSP90 Accumulation Promotes Vascular Remodeling in Pulmonary Arterial Hypertension. Am J Respir Crit Care Med 2018;198:90-103.
10. Bourgeois A, Bonnet S, Breuils-Bonnet S et al. Inhibition of CHK 1 (Checkpoint Kinase 1) Elicits Therapeutic Effects in Pulmonary Arterial Hypertension. Arterioscler Thromb Vasc Biol 2019;39:1667-1681.
11. Vitry G, Paulin R, Grobs Y et al. Oxidized DNA Precursors Cleanup by NUDT1 Contributes to Vascular Remodeling in Pulmonary Arterial Hypertension. Am J Respir Crit Care Med 2021;203:614-627.
